# FtsZ of Filamentous, Heterocyst-Forming Cyanobacteria Has a Conserved N-Terminal Peptide Required for Normal FtsZ Polymerization and Cell Division

**DOI:** 10.3389/fmicb.2018.02260

**Published:** 2018-10-02

**Authors:** Laura Corrales-Guerrero, Sergio Camargo, Ana Valladares, Silvia Picossi, Ignacio Luque, Jesús A. G. Ochoa de Alda, Antonia Herrero

**Affiliations:** ^1^Instituto de Bioquímica Vegetal y Fotosíntesis, Consejo Superior de Investigaciones Científicas and Universidad de Sevilla, Seville, Spain; ^2^Facultad de Formación del Profesorado, Universidad de Extremadura, Cáceres, Spain

**Keywords:** *Anabaena*, bacterial multicellularity, cell division, cyanobacterial FtsZ phylogeny, ZipN phylogeny

## Abstract

Filamentous cyanobacteria grow by intercalary cell division, which should involve distinct steps compared to those producing separate daughter cells. The N-terminal region of FtsZ is highly conserved in the clade of filamentous cyanobacteria capable of cell differentiation. A derivative of the model strain *Anabaena* sp. PCC 7120 expressing only an FtsZ lacking the amino acids 2–51 of the N-terminal peptide (ΔN-FtsZ) could not be segregated. Strain CSL110 expresses both ΔN-FtsZ, from the endogenous *ftsZ* gene promoter, and the native FtsZ from a synthetic regulated promoter. Under conditions of ΔN-FtsZ predominance, cells of strain CSL110 progressively enlarge, reflecting reduced cell division, and show instances of asymmetric cell division and aberrant Z-structures notably differing from the Z-ring formed by FtsZ in the wild type. In bacterial 2-hybrid assays FtsZ interacted with ΔN-FtsZ. However, ΔN-FtsZ-GFP appeared impaired for incorporation into Z-rings when expressed together with FtsZ. FtsZ, but not ΔN-FtsZ, interacted with the essential protein SepF. Both FtsZ and ΔN-FtsZ polymerize *in vitro* exhibiting comparable GTPase activities. However, filaments of FtsZ show a distinct curling forming toroids, whereas ΔN-FtsZ form thick bundles of straight filaments. Thus, the N-terminal FtsZ sequence appears to contribute to a distinct FtsZ polymerization mode that is essential for cell division and division plane location in *Anabaena*.

## Introduction

In the vast majority of bacteria and archaea, FtsZ protein polymerization underneath the cytoplasmic membrane is the event initiating cell division. With few exceptions, FtsZ is present and well conserved in most archaea, bacteria and chloroplasts. FtsZ is related to eukaryotic tubulin and polymerizes into a ring of bundled short filaments, the Z-ring, in the cytoplasmic side of the inner membrane at the future site of division (Fu et al., 2010; Erickson et al., 2010; Rowlett and Margolin, 2014; Szwedziak et al., 2014; Buss et al., 2015; Haeusser and Margolin, 2016). The Z-ring represents a scaffold for the recruitment of other components of the multiprotein complex called the divisome that expands the cell envelope and eventually effects membrane fission and cytoplasm and periplasm compartmentalization, thus leading to separation of two viable daughter cells. Besides its role at the initiation of divisome assembly, the Z-ring itself may contribute force for cytoplasmic membrane constriction during daughter cell separation (Li et al., 2007; Lutkenhaus et al., 2012; Coltharp et al., 2016). Recently, it has been proposed that directional movement of FtsZ filaments around the division ring by treadmilling guides the motion of septal cell wall synthesis enzymes, thus enabling correct envelope constriction and polar morphology (Bisson-Filho et al., 2017; Wagstaff et al., 2017; Yang et al., 2017). FtsZ polymerization is a dynamic process responding to GTP binding and hydrolysis, and in the model bacteria *Escherichia coli* and *Bacillus subtilis* a number of proteins influencing FtsZ polymerization and tethering of FtsZ to the plasma membrane, as well as restricting the localization of the Z-ring to midcell, have been thoroughly characterized (see Huang et al., 2013; Männik and Bailey, 2015). Moreover, in recent years a number of new regulators of FtsZ assembly have been described in other bacteria, showing that a diversity of mechanisms for FtsZ-ring dynamics may operate outside the well-studied bacterial models (e.g., Thanbichler and Shapiro, 2006; Treuner-Lange et al., 2013; Fleurie et al., 2014; Bisson-Filho et al., 2015; Holečková et al., 2015).

Cyanobacteria are a phylogenetically coherent group of bacteria characterized by their dominant photoautotrophic physiology (Flores and Herrero, 2014). Besides their significant contribution to the primary productivity in the modern oceans, the evolutionary relevance of cyanobacteria is maximum as the organisms that developed oxygenic photosynthesis leading to the Proterozoic oxidation of the Earth’s atmosphere (Schirrmeister et al., 2015) and as the ancestors, via endosymbiosis, of all the plastids nowadays present in algae and plants (Ochoa de Alda et al., 2014; Ku et al., 2015). The great morphological diversity of the cyanobacteria as a group (Rippka et al., 1979; Flores and Herrero, 2014) makes them attractive for comparative studies on cell division mechanisms. Especially appealing is the multicellular organization of filamentous cyanobacteria, a major evolutionary innovation (Schirrmeister et al., 2015; Herrero et al., 2016). In the model strain *Anabaena* sp. PCC 7120 (hereafter *Anabaena*), the organismic unit is a filament of interconnected cells that can be hundreds of cells long and that, depending on environmental conditions, could include different cell types specialized in specific physiological tasks (Flores and Herrero, 2010). As an example, heterocysts are cells specialized for the fixation of atmospheric nitrogen that are formed under conditions of combined nitrogen scarcity (Peterson and Wolk, 1978). In the *Anabaena* filament, cell-to-cell transfer of molecules with nutritional or regulatory function takes place (Flores and Herrero, 2010; Herrero et al., 2016). From the point of view of the cell envelope, cyanobacteria are diderm bacteria. In filamentous forms, whereas the inner membrane and peptidoglycan layers surround each cell, the outer membrane is continuous along the filament defining a shared periplasm (Mariscal et al., 2007). In addition, septal proteinaceous channels that connect contiguous cells contribute to cell–cell adhesion and molecular exchange (Flores et al., 2016). The conformation of a filament of interconnected bacterial cells invokes the participation of specific mechanisms during cell division that should differ from those of the common bacteria producing separated daughter cells.

Regarding cell division genes, cyanobacteria include some homologs of genes of both Gram-positive and Gram-negative bacteria, as well as some specific genes (Cassier-Chauvat and Chauvat, 2014; Herrero et al., 2016). Notably, genes encoding the common FtsZ tethers to the inner membrane FtsA and ZipA could be recognized neither in cyanobacterial genomes nor in plastid or nuclear genomes of plants and algae. However, some strains bear homologs of *sepF* that in *B. subtilis* influences the alignment of FtsZ filaments and represents an additional FtsZ tether (Duman et al., 2013). In the unicellular cyanobacterium *Synechocystis* sp. PCC 6803, SepF is indispensable and influences the formation of FtsZ polymers *in vitro* (Marbouty et al., 2009a). Also in *Synechocystis*, the cyanobacterial-specific factor ZipN has been suggested to play a central role in divisome assembly reminiscent of FtsA (Marbouty et al., 2009b).

Regarding *ftsZ*, homologs have been found in all available cyanobacterial genomes. Similar to its orthologs, the primary structure of the FtsZ proteins from cyanobacteria can be divided into four regions: an N-terminal peptide of variable length, a highly conserved globular core, a variable unstructured spacer, and the C-terminal conserved peptide (Vaughan et al., 2004). A wealth of studies on FtsZ from a variety of bacteria and archaea has provided detailed insight into the central region, which contain the determinants for GTP binding and hydrolysis, and the C-terminal region, which includes the sites of interaction of the most common FtsZ partners (Erickson et al., 2010; Buske and Levin, 2013; Du et al., 2015). However, no information about the possible role of the N-terminal peptide is available, except for a possible involvement in targeting to the organelle in the case of the *Arabidopsis* chloroplastic FtsZ2 variant (Fujiwara and Yoshida, 2001). This region is not conserved in length (it extends from a few to ca. 200 amino acids) or sequence between different taxonomic divisions and, in general terms, this part has received little attention, perhaps because it is very short in *E. coli* and *B. subtilis* (see e.g., Huang et al., 2013).

Within the framework of deciphering the distinct features of cell division in filamentous cyanobacteria, we have performed sequence comparisons of FtsZ through the cyanobacterial phylum. We have found that in heterocyst-forming strains, the conserved GTP-binding and hydrolysing core is preceded by a sequence of ca. 60 amino acids that is highly conserved within this clade. We have addressed the involvement of this N-terminal peptide in FtsZ polymerization and cell division in *Anabaena*.

## Materials and Methods

### Strains and Growth Conditions

*Anabaena* (also known as *Nostoc*) sp. PCC 7120 and strains CSFR18, CSL110, CSSC18, and CSSC19 were grown in BG11 medium (containing nitrate) (Rippka et al., 1979) or in BG11_0_ plus ammonium medium (containing 4–6 mM NH_4_Cl and 8–12 mM TES-NaOH buffer pH 7.5 instead of NaNO_3_), containing ferric citrate instead of ferric ammonium citrate, incubated at 30°C in the light (30 μE m^-2^ ⋅ s^-1^ from led lamps), in shaken liquid cultures or in plates in medium solidified with 1% Difco agar. For the mutants, media were supplemented with spectinomycin (Sp) and streptomycin (Sm) at 5 μg ⋅ ml^-1^ each in solid media or 2 μg ⋅ ml^-1^ each in liquid media (strains CSFR18, CSSC18 and CSSC19) or Sp and neomycin (Nm) at 20 μg ⋅ ml^-1^ in solid media or 10 μg ⋅ ml^-1^ in liquid media (strain CSL110). *E. coli* strains used for plasmid constructions, conjugation with *Anabaena* and BACTH analysis were as described previously (Ramos-León et al., 2015).

### Plasmid and Strain Constructions

DNA from *Anabaena* was isolated by the method of Cai and Wolk (1990). Plasmid vector pCSBN1 was based on pCSV3 (Valladares et al., 2011), after incorporation of a Nm^R^ determinant and the *sacB* gene (which determines sensitivity to sucrose) from pRL278 (Black et al., 1993) substituting for the C.S3 gene cassette. pCSL127 was generated by insertion into pCSBN1 of a DNA fragment of the *Anabaena ftsZ* genomic region extending from position -969 to +834 with regard to the ORF start, and lacking codons 2–51 of the ORF. The insert was generated with SacI ends by overlapping PCR with primers ftsZ-15/ftsZ-16 and ftsZ-17/fts-Z18 (all oligodeoxynucleotide primers are described in **Supplementary Table S1**) and was corroborated by sequencing. Plasmid pCSL127 was transferred by conjugation (Elhai et al., 1997) to strain PCC 7120 with selection for Nm^R^. Cultures of Nm^R^ exconjugants that had inserted the plasmid into the *ftsZ* locus by single crossover were used for selection of clones resistant to 5% sucrose which, by a second crossover event, would have substituted the native *ftsZ* gene by a version lacking codons 2–51 (Δ*5*′*-ftsZ*). However, despite repeated attempts, which included several rounds of sonication, to shorten the filaments, and growth in the absence of Nm, such chromosome versions could not be segregated.

Plasmid pCSL145 is based on pCSV3 with gene-cassette C.S3 substituted by C.K1 (Elhai and Wolk, 1988) and the bidirectional transcriptional terminators from gene-cassette C.S3 inserted at both sides of the plasmid polylinker (into the EcoRI and the SacI sites). pCSL145 includes a fragment of DNA of the *Anabaena ftsZ* genomic region extending from -969 bp to +1287 with regard to the start codon, but lacking the 2–51 codons of the ORF, inserted with KpnI ends, which was generated by overlapping PCR with primers ftsZ-19/ftsZ-16 and ftsZ-17/ftsZ-20. The insert of pCSL145 was corroborated by sequencing. Plasmid pCSL145 was transferred by conjugation to strain CSFR18 with selection for Nm^R^. One clone that, as indicated by PCR analysis (**Supplementary Figure S6**), bore in all its chromosome copies a native version of *ftsZ* expressed from the P_ND_ promoter (already present in CSFR18) and the Δ*5*′*-ftsZ* gene expressed from the native P*_ftsZ_* was selected and named strain CSL110. PCR analysis in strain CSL110 also indicated that the two possible arrays of the native and deleted genes were present in different chromosome copies (not shown).

Plasmid pCSE185 is a derivative of mobilizable vector pRL277 (Black et al., 1993) including the *gfp-mut2* gene. Plasmid pCSSC38 includes an *Anabaena* DNA fragment encompassing the *ftsZ* gene and 996 bp of upstream sequences cloned in the pSpark^®^ plasmid vector. Plasmids pCSSC38 and pCSL145 were used as template for PCR with primer pairs ftsZ-36/ftsZ-37 and ftsZ-38/ftsZ-39, respectively. The amplification products were cloned, with SacI/NheI ends, into pCSE185, generating plasmids pCSSC39 and pCSSC36, respectively. In plasmid pCSSC39 the *mut2-gfp* gene, preceded by four Gly-encoding codons, is fused to the last amino acid-encoding codon of the *ftsZ* ORF; in plasmid pCSSC36 the *mut2-gfp* gene, preceded by four Gly-encoding codons, is fused to the last amino acid-encoding codon of the Δ*5*′*-ftsZ* ORF. Plasmids pCSSC39 and pCSSC36 were transferred to *Anabaena* by conjugation. Clones resistant to Sm and Sp were selected. The genetic structure of exconjugants was verified by PCR (not shown). A clone that received pCSSC39 and included the *ftsZ-mut2gfp* gene was selected and named strain CSSC19, and a clone that received pCSSC36 and included the Δ*5*′*-ftsZ-mut2gfp* gene was selected and named strain CSSC18 (**Figure 11A**).

To express in *E. coli* the *Anabaena ftsZ* and the Δ*5*′-*ftsZ* genes preceded by a sequence encoding a 6His-tag, PCR was performed using plasmid pCSFR22 (Ramos-León et al., 2015) as template and primer pairs ftsZ-8/ftsZ-43 and ftsZ-8/ftsZ44, respectively. The products were cloned in vector PROEX-HTb, producing plasmids pCSAV264 and pCSAV265, respectively, which were corroborated by sequencing and transferred to *E. coli* strain XL1Blue.

### Analysis of *ftsZ* Expression by RT-qPCR and Semi-Quantitative RT-PCR

RNA was isolated as described previously (Mohamed and Jansson, 1989) from filaments of *Anabaena* strains grown in BG11 medium and incubated for 5 days in BG11 or BG11_0_ plus ammonium medium (supplemented with antibiotics for the mutants). RNA (248 ng) was used for reverse transcription with the Quantitec Reverse Transcription kit (Qiagen) as described in Ramos-León et al. (2015), but using the following amplification protocol: one cycle at 95°C for 3 min; 30 cycles of: 95°C for 10 s, 62°C for 20 s and 72°C for 20 s. In RT-qPCR, the relative transcript levels of *ftsZ* (*alr3858*) and Δ*5*′-*ftsZ* are expressed as the ΔΔ*C*t value using genes *alr0599* and *all5167* (see Flaherty et al., 2011) for normalization. The primer pairs used were: alr0599-1/alr0599-2 (*alr0599*), all5167-1/all5167-2 (*all5167*), ftsZ-24/ftsZ-25 (Δ5′-*ftsZ*) and ftsZ-28/ftsZ-29 (*ftsZ*), respectively. In semi-quantitative RT-PCR the primer pair used was ftsZ-47/ftsZ-48, and the gene *alr0599* was used for normalization. The number of cycles at which the PCR reaction was in the exponential range was empirically determined. Samples were taken and resolved by electrophoresis in agarose gels, and the Image Lab software (Bio-Rad) was used for quantification.

### Preparation of *Anabaena* Cell-Free Extracts and Western Blot Analysis

Whole filament suspensions in buffer A (50 mM Tris-HCl [pH 8.0], 200 mM NaCl, 10% glycerol) were supplemented with a protease inhibitor mixture tablet (*c*Omplete Tablets, Mini EDTA-free; Roche) and passed through a French pressure cell at 20,000 psi three times. Cell debris were removed by centrifugation at 16,100 × *g* (4°C, 10 min). Immunoblotting and probing with polyclonal antibodies generated in rabbits against purified 6His-tagged *Anabaena* FtsZ protein (Ramos-León et al., 2015) or, as a loading control, against amino acids 206–219 of the EF-Tu factor (All4337) were performed by standard procedures.

### FtsZ and ΔN-FtsZ Purification

6His-FtsZ and 6His-ΔN-FtsZ were obtained from *E. coli* bearing plasmids pCSAV264 and pCSAV265, respectively (see above), after induction with isopropyl-β-D-1-thiogalactopyranoside (IPTG). Proteins were purified by chromatography through a 1 ml His-select column from GE Healthcare, using imidazole to elute the retained proteins and, after filtration through GE Healthcare PD-10 columns (Sephadex G-25 M), were finally dissolved in a buffer containing 50 mM HEPES (pH 7.2), 50 mM KCl, 5% glycerol.

To remove the 6His-tag, purified 6His-FtsZ and 6His-ΔN-FtsZ proteins were digested with AcTEV protease (Invitrogen), at 0.5 enzyme units/μg protein, 25°C overnight in the buffer provided by the manufacturer. After that, samples were dialyzed for 4 h, at 4°C, against 50 mM HEPES (pH 7.2), 50 mM KCl, 5% glycerol, to remove DTT and EDTA present in the digestion buffer. Then, FtsZ and ΔN-FtsZ proteins were again filtered through Ni^2+^-resins that retain the AcTEV protease and His-tagged proteins. For proteins to be used for GTPase activity assays, incubation with protease was performed at 4°C overnight followed by dialysis for 1 h, and the final filtration through Ni^2+^-resin was omitted (by SDS-PAGE, it was assessed that more than 95% protein lacked the 6His tag).

### FtsZ Polymerization Assays

For polymerization assays, 3 μM protein was incubated in polymerization buffer (50 mM HEPES [pH 7.2], 50 mM KCl, 5% glycerol) supplemented with 10 mM MgCl_2_ and 2 mM GTP at 30°C for the time indicated, and then centrifuged at 25°C in a Beckman XL80 ultracentrifuge equipped with rotor 42.2Ti at 150,234 *g* for 15 min. The supernatant was decanted without disturbing the pellet, and the sediment was dissolved in SDS-loading buffer. Aliquots of the sediment and supernatant fractions were run in 10% SDS-PAGE gels. After scanning, the intensity of the bands was estimated using the ImageJ 1.47i software^1^.

### GTPase Activity

3 μM purified protein was incubated in polymerization buffer (50 mM HEPES [pH 7.2], 50 mM KCl, 5% glycerol) supplemented with 10 mM MgCl_2_, at 30°C for 20 min. Then, 2 mM GTP was added, and aliquots of the reaction mixture were withdrawn for determination of released Pi according to Gawronski and Benson (2004). For each time point, the corresponding value of a control mixture without protein incubated under the same conditions was subtracted.

### BACTH Assays

BACTH assays based on the reconstitution of adenylate cyclase from *Bordetella pertussis* (Karimova et al., 2005) were performed. All tested genes were amplified by PCR using plasmid pCSFR22 (Ramos-León et al., 2015) (*ftsZ* and Δ5′-*ftsZ*) or *Anabaena* genomic DNA (*zipN*, *sepF*, *minC*) as template. The following primer pairs were used for amplification: ftsZ-46/ ftsZ-45 (FtsZ-T18), ftsZ-33/ftsZ-45 (ΔN-FtsZ-T18, ΔN-FtsZ-T25), ftsZ-34/ftsZ-32 (T18-FtsZ), ftsZ-33/ftsZ-32 (T18-ΔN-FtsZ), ftsZ-35/ ftsZ-32 (T25-FtsZ), ftsZ-31/ftsZ-32 (T25-ΔN-FtsZ), sepF-18/ sepF-21 (SepF-T18), sepF-18/sepF-17 (T18-SepF), sepF-18/sepF-21 (SepF-T25), sepF-16/sepF-17 (T25-SepF), zipN-21/zipN-22 (T25-ZipN), minC-1/minC-4 (MinC-T18, MinC-T25), minC-1/minC-3 (T18-MinC), and minC-2/minC-3 (T25-MinC). The resulting PCR products, which were flanked by EcoRI and PstI ends (for *ftsZ*, Δ5′-*ftsZ*, and *sepF*), or KpnI and PstI ends (*zipN* and *minC*), were cloned in pUT18, pUT18C, pKNT25, or pKT25 digested with the same enzymes, producing fusions to the 5′ or 3′ ends of the genes encoding the adenylate cyclase T18 and T25 fragments. All the resulting plasmids were verified by sequencing. Fusions of the *ftsZ* gene to the 5′ end of T25 were as previously described (Ramos-León et al., 2015). Plasmids were transformed into *E. coli* XL1-Blue for amplification. Isolated plasmids were co-transformed into strain BTH101 (*cya*-99), and the transformants were plated on solid LB medium containing selective antibiotics and 1% glucose.

To estimate the strength of interactions, β-galactosidase activity was measured after growth in liquid medium in the presence of IPTG and antibiotics, using *o*-nitrophenol-β-galactoside as a substrate. The *o*-nitrophenol produced per mg of protein versus time was plotted, and β-galactosidase activity was calculated from the slope of the linear function.

### Microscopy

For staining with FM1-43, 2.5 μl of the dye (0.1 mg/ml DMSO; Molecular Probes) were added to 0.1 ml of cell suspension, incubated for 10 min at room temperature and washed twice with growth medium. For staining with DAPI, 5.0 μl of the dye (10 μg/ml) were added to 0.1 ml of cell suspension. For immunolocalization of FtsZ, filaments were treated as described (Ramos-León et al., 2015). Samples were visualized in a Leica DM6000B fluorescence microscope and photographed with an ORCA-ER camera (Hamamatsu). Fluorescence was monitored with a FITC L5 filter (excitation, band-pass 480/40 filter; emission, band pass 527/30 filter for FM1-43 staining and immunolocalization; excitation 360/40 and emission 470/40 for DAPI staining). Images were treated with the Leica Application Suite Advanced Fluorescence software and with ImageJ 1.47i software. Cell area was estimated automatically with ImageJ 1.47i processing of light-microscopy images, and the obtained results were compared to those obtained by manual estimations with fluorescence microscopy images after staining filaments with the FM1-43 dye. Data were plotted using the open source software RStudio Desktop^2^. Nucleoid area was estimated with ImageJ 1.47i processing of DAPI images. GFP fluorescence and *Anabaena* autofluorescence were monitored with a Leica HCX PLAN-APO 63× 1.4 NA oil immersion objective attached to a Leica TCS SP2 confocal laser-scanning microscope. Excitation was made using 488 nm irradiation from an argon ion laser, and fluorescence was collected across windows of 500–540 nm (GFP imaging) or 630–700 nm (cyanobacterial autofluorescence). ImageJ 1.47i software was used for analysis and graphical representation of fluorescence data.

For visualization of FtsZ filaments by electron microscopy, 3 μM protein was incubated for 20 min at 30°C in polymerization buffer (see above) supplemented with 10 mM MgCl_2_ and 2 mM GTP, after which 10 μl aliquots were placed on a 300-mesh carbon-coated copper grid (CF300-Cu from Aname) for 2 min, blotted dry, stained with 1% uranyl acetate for 5 min and blotted dry again. Grids were observed and photographed with a Libra 120 (Zeiss) transmission electron microscope.

### Bioinformatics

Remote homologs of the N-terminal sequence of *Anabaena* FtsZ were detected by using PSI-BLAST search against RefSeq database until convergence (7 iterations). Out of 215 genomes available (as in November 2016), a representative set of 96 was selected to avoid overrepresented species such as *Microcystis aeruginosa*, *Planktothrix agardhii*, marine *Prochlorococcus* and *Synechococcus*. Sequences from the genome of *Gloeomargarita lithophora* were added later (September 2017). Most FtsZ and MinC sequences were retrieved from NCBI-Refseq (Pruitt et al., 2012) and IMG (Markowitz et al., 2014) databases, except the FtsZ sequence of *Neosynechococcus sphagnicola* that was retrieved from its genome-project web page after joining two contigs. Each *ftsZ* sequence was inspected for alternative translation start point and reassigned, if required, using IMG viewer. ZipN sequences from 146 representative cyanobacterial genomes were retrieved in September 2017. FtsZ, MinC and ZipN sequences were aligned using MAFFT (Katoh and Standley, 2013) whereas the phylogenetic trees were reconstructed using Phyml 3.1 (Guindon et al., 2010), after selection of best evolutionary model with Prottest 3.4 (Darriba et al., 2011).

Guide tree for the evolution of cyanobacteria (those species retrieved for FtsZ and MinC analysis) was based on the phylogeny of concatenated small and large rRNA sequences. These sequences were retrieved, aligned and concatenated as previously described (Santamaría-Gómez et al., 2016). One exception was the 23S rRNA gene of *G. lithophora*, in which two endonuclease-encoding group I introns were removed before alignment.

In order to estimate the average relative contribution of cyanobacterial lineages to the divergence of protein sequences, a phylogenetic tree from the concatenation of 97 proteins isolated from 102 strains (Ponce-Toledo et al., 2017) was constructed. The concatenated data set (kindly provided by David Moreira) was trimmed and the constant sites removed before phylogenetic reconstructions (Ochoa de Alda et al., 2014) using model LG+CAT in RAxML (Stamatakis, 2014). This average relative contribution to protein divergence was also estimated from published phylogenetic trees obtained after concatenation of a reduced number of proteins and different taxa: 31 conserved proteins from 126 taxa (Shih et al., 2013) and 23 conserved proteins from 191 taxa (Mareš, 2017). This diversity in proteins and taxa reinforce the robustness of our analysis of protein change during lineage expansion.

The relative contribution of the expansion of a lineage to the divergence of protein sequences was estimated as follows: over a printed phylogenetic tree (either from FtsZ, MinC, ZipN, or concatenated proteins phylogenies), we first measured the distances from a reference node (the branching point of Yellowstone *Synechococcus* strains, which is present in all trees) to the tips corresponding to a given lineage (reference distances). Then, we measured the distance from the reference node to the node clustering the lineage. The subtraction of this distance from the reference distances corresponds to the genetic distances accumulated for the strains during lineage expansion (lineage distances). The relative contribution of a lineage expansion to protein sequence divergence is the ratio (lineage distances)/(reference distances).

PATRISTIC (Fourment and Gibbs, 2006) was used to infer genetic distances (substitutions per site) from phylogenetic trees of concatenated rRNAs (K*_rRNA_*), FtsZ (K*_FtsZ_*), as well as its core and variable regions. Genetic distances were calculated from *Gloeobacter* to each tip before the incorporation of *G. lithophora* to the analysis.

## Results

### Analysis of Cyanobacterial FtsZ Sequences

**FIGURE 1:**
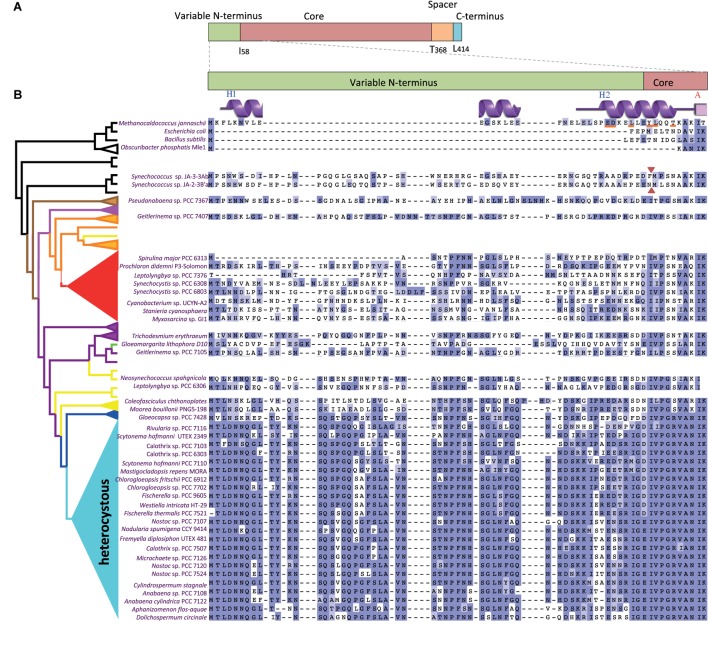
Sequence comparisons of the FtsZ variable N-terminal peptide in a representative set of cyanobacteria. **(A)** FtsZ regions (above) and amino acid boundaries (below) in *Anabaena* sp. PCC 7120. **(B)** Multiple alignment of the N-termini of FtsZ (enlarged region) from a subset of model organisms (top), melainabacteria (*Obscuribacter*) and cyanobacteria. The represented heterocystous cyanobacterial strains were chosen by their higher disparity, whereas a *Pseudanabaena* sp. was included by its high genetic distance to the clade of heterocystous strains. A simplified FtsZ tree (left) identifies the clade to which they belong. Red arrowheads indicate the first amino acids of the FtsZ sequence of two *Synechococcus* sp. JA strains before re-annotation. Gaps are indicated by dashes. A residue is colored dark blue if it matches the consensus sequence at that position. If it does not match the consensus residue but the 2 residues have a positive Blosum62 score, it is colored light blue (see **Supplementary Figure S1**). Features of the secondary structure of *Methanocaldococcus* FtsZ, PDBsum entry 1W59, (Oliva et al., 2004) are shown over its sequence and the residues involved in FtsZ dimerization are underlined in red.

FtsZ from *Anabaena* contains the four regions previously identified in other orthologs but, in contrast to other well-characterized FtsZs such as those of *Methanocaldococcus jannaschii*, *B. subtilis*, and *E. coli*, the N-terminal variable region is much longer (**Figure 1**). To characterize the sequence and distribution of this region, a PSI-BLAST search of remote homologs against the RefSeq database was performed using the N-terminal region of *Anabaena* (gi| 499307224, region: 1.60) as query. Results showed that (i) homologs were only present in the phylum *Cyanobacteria*; (ii) homologous sequences were always appended as an N-terminal region of FtsZ; and (iii) this sequence likely originated early during the diversification of cyanobacteria since it is observed in *Pseudanabaena* strains, which branch deeply in the phylum.

To reinforce the search of FtsZ N-terminal sequences that are homologous to that of *Anabaena*, we retrieved 216 cyanobacterial sequences annotated as *ftsZ* in GenBank noting that: (i) many lacked a sequence encoding an N-terminal variable region (such as those of the early divergent *Synechococcus* sp. JA strains), and (ii) some closely related strains, such as strains of *Spirulina* sp., differed in the length of the variable region. This prompted us to revise the assignment of the *ftsZ* translation start point in a representative and diverse set of 97 cyanobacterial genomes. From this, we identified 20 presumable misannotations where sequences encoding putative N-terminal extensions were excluded, likely because of lack of similarity to FtsZ sequences from model organisms. In this case, the misannotation rate considerably exceeded the 10% estimated for the automatic prediction of protein coding sequences (Markowitz et al., 2014). This illustrates the difficulty in annotating variable N-termini, an obligate step to explore a potential function for these sequences. **Supplementary Figure S1** presents the results of the sequence alignment of FtsZ proteins from these 97 strains (a representative set of strains is shown in **Figure 1B**). With the exception of one strain (*Synechococcus* sp. PCC 6312), in which it is totally missing, the length of the FtsZ N-terminal part in cyanobacteria varies between 20 and more than 80 amino acids. In comparison to the core region, the N-terminal peptide is highly divergent within this phylum, with few conserved residues (**Supplementary Figure S1**). However, alignment of subsets of sequences revealed that the N-terminal peptide of FtsZ is well conserved within some clades of filamentous cyanobacteria, with a particularly high degree of conservation in filamentous heterocyst-forming strains (**Figure 1B**).

### Evolution of FtsZ in the Cyanobacterial Phylum

**FIGURE 2:**
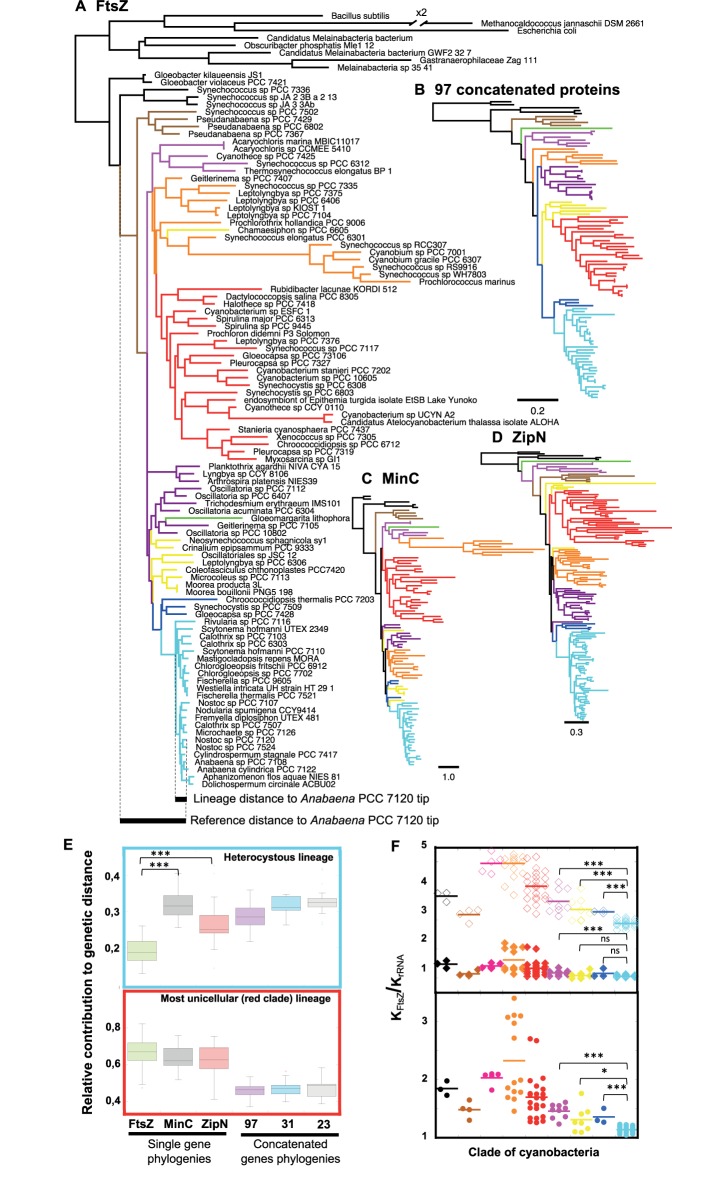
Phylogenetic analysis of FtsZ in a representative set of cyanobacteria. **(A)** Phylogenetic reconstruction of 97 FtsZs (top) and example of estimation of the reference distance and lineage distance to *Anabaena* PCC 7120 (referred in the tree as *Nostoc* sp. PCC 7120.) (Bottom). The // symbol indicates a branch that has been graphically reduced to 50% of its original length. **(B)** Guide tree for the evolution of 102 cyanobacterial strains based on the concatenation of 97 proteins. **(C)** Phylogenetic tree of 97 MinC proteins. **(D)** Phylogenetic tree of 146 ZipN proteins. Scales represent genetic distances. **(E)** Relative contribution of the expansion of heterocystous cyanobacteria (top) and red clade (bottom) lineages to the divergence of protein sequences. These contributions were estimated from FtsZ, MinC, and ZipN phylogenies (**A,C,D** trees) and trees obtained after concatenation of 97 **(B)**, 31 and 23 proteins (see section “Materials and Methods”). The horizontal line inside the box is the median, and the top and bottom are the 25th and 75th percentiles. The top and bottom ends of the vertical lines approximate the 95th and 5th percentiles, respectively. **(F)** FtsZ vs. rRNA ratio of relative genetic distances (K*_FtsZ_*/K*_rRNA_*) for each clade of cyanobacteria (bottom) and relative genetic distances (K*_FtsZ_*/K*_rRNA_*) for the core (top, filled diamonds) and the variable N-terminal peptide (top, open diamonds) regions of FtsZ in each clade. Branches and dots are colored according to the clade they belong in the guide tree (**Supplementary Figure S3**). A horizontal straight bar marks the average value for each clade. Connecting lines are used to express significance of Wilcoxon–Mann–Whitney test: ^∗∗∗^*P* < 0.0001, ^∗^*P* < 0.05; ns (not significant at the 0.05 level). **Supplementary Figures S2**–**S5** provide additional information of all trees.

A phylogenetic tree based on full FtsZ sequences from cyanobacterial species, some Melainabacteria and some well characterized model strains (as root) was constructed (**Figure 2A**). This tree mirrored almost perfectly the reference trees for cyanobacterial phylogeny based on the concatenation of proteins (**Figure 2B** and **Supplementary Figure S2**) or on 16S-23S rRNA sequences (**Supplementary Figure S3**), as well as other phylogenies recently published (Shih et al., 2013; Mareš, 2017; Ponce-Toledo et al., 2017) (see **Supplementary Text** for more details). This suggests that FtsZ was mostly vertically inherited within this phylum, which is consistent with the difficulty for its horizontal transfer described for other prokaryotes (Sorek et al., 2007). A visible difference between FtsZ and reference phylogenies was the shorter relative length of branches corresponding to heterocystous cyanobacteria (pale blue cluster in **Figure 2A**). This difference in the relative length of branches was also observed when the FtsZ tree was compared to trees for other cell division proteins such as MinC (**Figure 2C** and **Supplementary Figure S4**) and ZipN (**Figure 2D** and **Supplementary Figure S5**). This conspicuous difference pointed to the existence of factors making FtsZ resistant to evolutionary change during the diversification of heterocystous cyanobacteria.

A quantitative assessment of this phenomenon was obtained by two different approaches: (i) the estimation of the relative contribution of the expansion of a lineage to FtsZ sequence divergence (an example, taking as a reference node the branching point of Yellowstone *Synechococcus* strains, is shown at the bottom of the FtsZ tree, **Figure 2A**), and (ii) the estimation of the relative genetic distances of FtsZ compared to 16-23S rRNA sequences, taking the latter as a reference for the pace of evolutionary change of the species (see section “Materials and Methods”).

In heterocyst-forming cyanobacteria (pale blue clade), the relative contribution of the expansion of the lineage to FtsZ divergence is significantly lower than that observed for the MinC or ZipN proteins (**Figure 2E** top). For these two proteins, the range of relative protein sequence divergence is close to a set of reference intervals calculated from phylogenetic trees based on the concatenation of 97 (**Figure 2B** and **Supplementary Figure S2**), 31 (Shih et al., 2013) or 23 (Mareš, 2017) representative cyanobacterial proteins. In order to ascertain whether the relative low divergence of FtsZ observed in heterocyst-forming cyanobacteria was specific of this lineage, we measured this relative contribution during the expansion of the red clade, which mostly comprises unicellular cyanobacteria. Values obtained for FtsZ were similar to those of MinC and ZipN, indicating that in the red clade the pace of evolution of FtsZ was similar to those of MinC and ZipN factors (**Figure 2E** bottom). These results support the existence of higher evolutionary constraints for FtsZ in the heterocystous linage, which is consistent with the short length of branches in the FtsZ-based tree.

Regarding relative genetic distances of FtsZ sequences, K*_FtsZ_*/K*_rRNA_*, compared to its sister group (dark blue cluster in **Figure 2**), this value was significantly lower for heterocyst-forming cyanobacteria (Wilcoxon–Mann–Whitney test, *p*-value = 0.0001), supporting higher evolutionary constraints for FtsZ in this clade (**Figure 2F** bottom). In addition, individual FtsZ regions were analyzed (**Figure 2F**, top). Although the N-terminal peptide evolved four times faster than the core, lower K*_FtsZ_*/K*_rRNA_* values were observed for the former in heterocyst-forming cyanobacteria than in sister clades (dark blue and yellow clades). In contrast, values for the core region were comparable to those of sister groups. This supported a specific conservation of the N-terminal part of FtsZ in heterocyst-forming cyanobacteria.

The prevalence of the N-terminal variable region in cyanobacteria, and furthermore its outstanding conservation in heterocystous strains, pointed to a functional role of this region not previously considered. In the following sections of this work, we experimentally address the role of the *Anabaena* FtsZ N-terminal peptide.

### FtsZ Polymerization Assays

**FIGURE 3:**
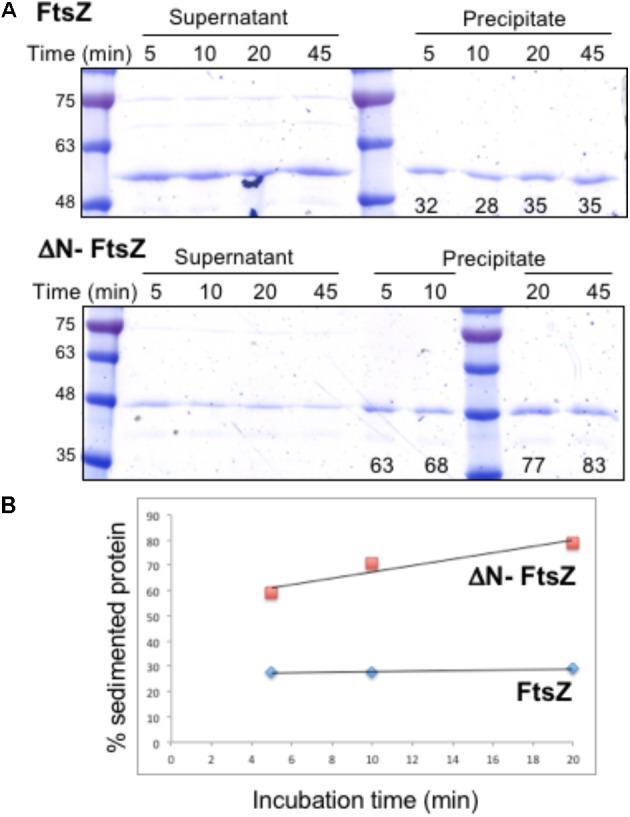
Polymerization of *Anabaena* FtsZ and ΔN-FtsZ proteins. **(A)** 3 μM of FtsZ or ΔN-FtsZ protein was incubated in the presence of 2 mM GTP under the conditions described in Section “Materials and Methods”. At the indicated times aliquots of the incubation mixtures were centrifuged at 150,234 *g* during 15 min. Aliquots of the resulting supernatant and precipitated fractions were resolved in SDS-PAGE gels, and bands were scanned and quantified. The numbers at the bottom of gels represent the % of total protein found in the sediment. Approximate size (kDa) correspondence of molecular weight markers is indicated at left. **(B)** The average values of two independent experiments with very similar results are represented.

First, we studied the role of the N-terminal peptide in polymer formation by *Anabaena* FtsZ. Purified *E. coli* FtsZ protein can polymerize *in vitro* in a GTP-dependent manner, and the extent of polymerization can be analyzed after sedimentation by centrifugation (Mukherjee and Lutkenhaus, 1998). We studied the capacity of *in vitro* polymerization of *Anabaena* FtsZ and ΔN-FtsZ (lacking amino acids 2–51 of the native protein). Proteins purified from *E. coli* bearing plasmid pCSAV264 or pCSAV265, respectively (see section “Materials and Methods”), were incubated in assay mixtures in the presence of GTP, and, after centrifugation, the protein content in the supernatant and precipitated fractions was analyzed by SDS-PAGE. To settle conditions for polymer sedimentation, we used different assay conditions including various protein concentrations, pH values and GTP concentrations, and different centrifugation speeds and duration (not shown). The selected conditions are described under Section “Materials and Methods.” **Figure 3** illustrates the results obtained after incubation of FtsZ and ΔN-FtsZ for different periods of time under those conditions. It shows that for FtsZ the percentage of sedimented protein was relatively low (ca. 30% of total) and constant, and that for any time-point the fraction of sedimented ΔN-FtsZ was considerably higher than that of FtsZ (**Figure 3**).

**FIGURE 4:**
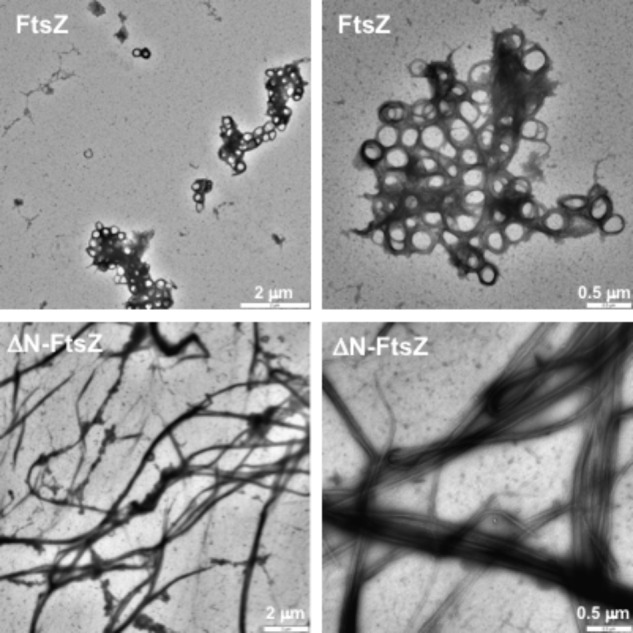
Polymers of *Anabaena* FtsZ and ΔN-FtsZ proteins. Proteins (3 μM) were incubated during 20 min in the presence of 2 mM GTP under polymerization conditions and visualized after negative staining with uranyl acetate. Magnification was: 3,150× **(upper left)**, 2,000× **(lower left)**, 8,000× **(right)**.

FtsZ polymers formed *in vitro* can be visualized by electron microscopy after negative staining with uranyl acetate. When both *Anabaena* FtsZ and ΔN-FtsZ proteins incubated under the above polymerization conditions were visualized by electron microscopy, we observed that FtsZ massively bent forming aggregates of loops (**Figure 4**, upper panels). In contrast, ΔN-FtsZ appeared to aggregate more intensively forming thick bundles of filaments (**Figure 4**, lower panels). The larger aggregates formed by ΔN-FtsZ could potentiate sedimentation, contributing to the higher fraction of protein that could be recovered in the precipitate after centrifugation.

Filaments of 6His-tagged proteins were also visualized. Filaments formed by 6His-ΔN-FtsZ (**Supplementary Figure S7**, lower panels) appeared very similar to those of ΔN-FtsZ. Regarding polymers formed by 6His-FtsZ, although resembling those by FtsZ in that they also appeared rather flexible and curly frequently forming hoops (**Supplementary Figure S7**, upper panels), in 6His-FtsZ the loops appeared as part of thin filaments that were not observed with FtsZ. Thus, it appears that in the case of FtsZ protein, the 6His-tag limits polymer looping.

### GTPase Activity

**FIGURE 5:**
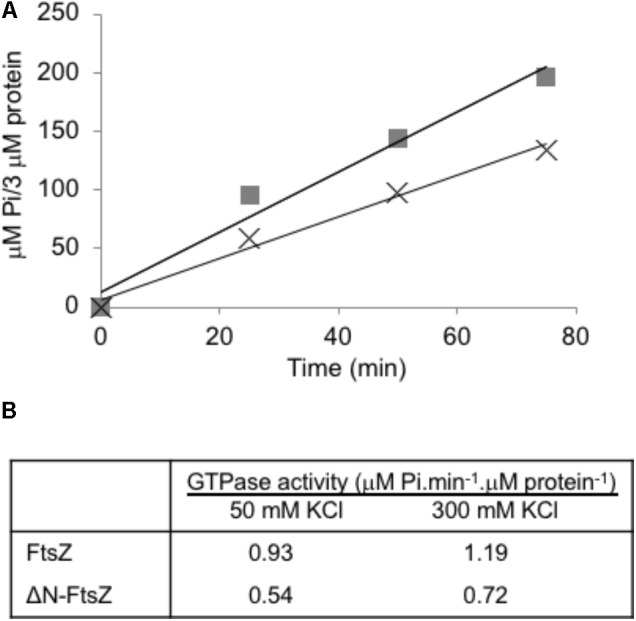
GTPase activity of *Anabaena* FtsZ and ΔN-FtsZ proteins. **(A)** GTPase activity was determined as specified in Section “Materials and Methods” with 3 μM of FtsZ (squares) or ΔN-FtsZ (crosses). At the times indicated, Pi produced was determined in aliquots of each reaction mixture. **(B)** GTPase activity was determined in reaction mixtures containing 3 μM protein and 50 or 300 mM KCl. Activity was calculated from the slope of the linear function as in **(A)**. Figures are the mean of two independent assays with very similar results.

Purified FtsZ protein from different sources exhibits GTPase activity, which requires protein polymerization (e.g., Mukherjee and Lutkenhaus, 1998; see Erickson et al., 2010). When *Anabaena* FtsZ was incubated under conditions similar to those used for polymerization assays, GTPase activity of ca. 1 μM GTP hydrolyzed⋅min^-1^ ⋅ μM FtsZ^-1^ was measured, and similar values were observed increasing the KCl concentration from 50 to 300 mM (**Figure 5**). ΔN-FtsZ protein also exhibited GTPase activity, which was ca. 0.6 times that of FtsZ (**Figure 5**). (See **Supplementary Figure S8** for data with His-tagged proteins).

### Generation of *Anabaena* Strains Expressing ΔN-FtsZ

**FIGURE 6:**
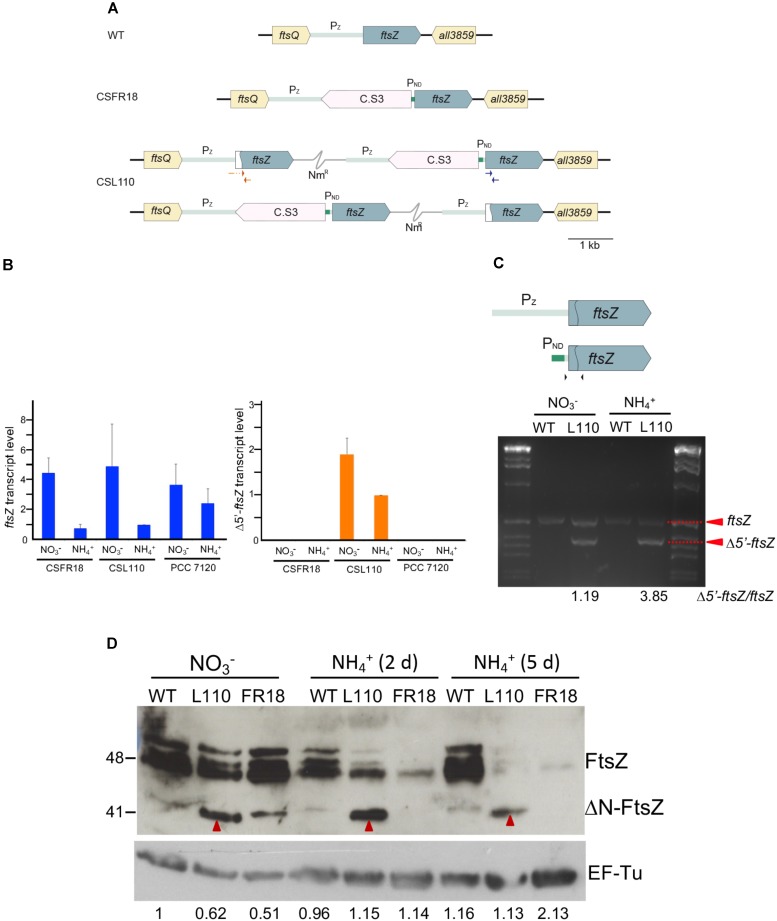
**(A)** Genomic structure and *ftsZ* expression in strain CSL110. Schematic of the *ftsZ* genomic region in *Anabaena* (WT) and strains CSFR18 (which expresses the native *ftsZ* gene from a nitrogen-regulated promoter) and CSL110 (which expresses the native *ftsZ* gene from a nitrogen-regulated promoter and the Δ*5*′*-ftsZ* gene from the *ftsZ* gene promoter). A white sector in the left part of a *ftsZ* ORF denotes a deletion of the 2nd to 52nd codons rendering allele Δ*5*′*-ftsZ*. Nm^R^ represents a Nm-resistance genetic determinant included in the plasmid inserted in the chromosome (thin gray trace). C.S3 represents a gene-cassette providing Sm^R^ and Sp^R^. P_ND_, nitrogen-regulated promoter; P_z_, *ftsZ* gene promoter. The two chromosome versions shown are present in strain CSL110 (see section “Materials and Methods” for details). **(B)** Levels of *ftsZ* and Δ*5*′*-ftsZ* transcripts in strains CSFR18, CSL110, and PCC 7120 (WT). RNA was isolated from BG11 (containing nitrate)-grown filaments incubated 5 days under culture conditions in either BG11 or BG11_0_ plus ammonium medium, as indicated, and RT-qPCR was performed as described in Section “Materials and Methods.” Relative values with regard to those of CSL110 incubated with ammonium are represented for each allele. The positions of the primers used are indicated in blue (for *ftsZ*) and red (for Δ*5*′*-ftsZ*) in **(A)**. Three or four independent cultures of each strain and incubation condition were used; error bars represent the standard error of the mean. **(C)**. Semi-quantitative RT-PCR was performed with the same cDNAs used for RT-qPCR. The primers used are indicated as black arrowheads over a scheme of the *ftsZ* gene as preceded by its native promoter (P_z_, gray) or the P_ND_ promoter (green), which is separated from the ORF by a 52-nucleotide stretch of the native region. The sequence deleted in Δ*5*′*-ftsZ* is marked by the curved line inside the ORF. The relative amounts of *ftsZ* (549 bp-band) and Δ*5*′*-ftsZ* (399 bp-band) transcripts are indicated at the bottom of figure. Size marker (markerX, Roche) is shown at left and right. **(D)** Western blot analysis of FtsZ. Filaments grown for 7 days in BG11 medium (${NO}_{3}^{-}$) or further incubated for 2 or 5 days in BG11_0_ plus ammonium medium (${NH}_{4}^{+}$) of *Anabaena* (WT) and strains CSL110 (L110) or CSFR18 (FR18) were used to prepare cell extracts. Aliquots of the resulting preparations were loaded into 15% SDS/PAGE gels, electrophoresed and probed with antibodies raised against the *Anabaena* FtsZ protein (Ramos-León et al., 2015) (upper panel). As a loading control, hybridization was also performed with an antibody against the EF-Tu factor (relative amounts of EF-Tu factor obtained after scanning and quantification are indicated) (lower panel). Red arrowheads point to a putative ΔN-FtsZ band (this band could be resolved from a slightly upper band produced by FtsZ, as shown in the WT and CSFR18). Size indicators are shown at the left. Predicted MW: FtsZ, 44.73 kDa; ΔN-FtsZ, 39.28 kDa; EF-Tu, 44.8 kDa.

To study the role of the N-terminal FtsZ peptide *in vivo*, we sought the generation of mutant derivatives of *Anabaena* expressing a FtsZ version lacking this sequence. A mutated version of *ftsZ* lacking the amino acids 2–51 of the N-terminal peptide (Δ*5*′*-ftsZ*, encoding ΔN-FtsZ) was introduced in *Anabaena*; however, despite repeated attempts, this mutation could not be segregated (see section “Materials and Methods” for details). Thus, we sought the generation of *Anabaena* derivatives conditionally expressing different proportions of the native FtsZ and ΔN-FtsZ proteins. We used as a parental strain the *Anabaena* mutant CSFR18 that expresses *ftsZ* from a synthetic regulated promoter (P_ND_) with low activity in medium supplemented with ammonium as the nitrogen source (Ramos-León et al., 2015). Using CSFR18 as a recipient of the Δ*5*′*-ftsZ* gene construct, strain CSL110 was generated that expresses, besides the native version of *ftsZ* from the P_ND_ promoter, the Δ*5*′*-ftsZ* allele from the native *ftsZ* promoter (**Figure 6A**; see Materials and Methods). RT-qPCR analysis (**Figure 6B**) showed that both gene versions, *ftsZ* and Δ*5*′*-ftsZ*, were expressed in strain CSL110. As described previously, strain CSFR18 expressed lowest levels of the native *ftsZ* gene in the presence of ammonium (as compared to expression in medium containing nitrate as the nitrogen source), which was also the case for CSL110. Consistently, in strain CSL110 the proportion of *ftsZ* to Δ*5*′*-ftsZ* expression was lowest in the presence of ammonium (e.g., the amount of Δ*5*′*-ftsZ* transcripts was ca. four times higher than that of *ftsZ* in the experiment shown in **Figure 6C**). Analysis by western blot with antibodies raised against *Anabaena* FtsZ showed several bands that would correspond to FtsZ (possibly including multimers and degradation products). Besides, a distinct band (approximately migrating as a molecule of 41 kDa in size) was produced by filaments of strain CSL110, but not by filaments of CSFR18 or the wild type (**Figure 6D**; see band marked with red arrowhead) (see also **Supplementary Figure S9**). This band should thus correspond to the ΔN-FtsZ protein (predicted size, 39.28 kDa). **Figure 6D** also shows that after incubation in ammonium-containing medium the amount of FtsZ became very low in CSFR18, and that the ΔN-FtsZ band became the predominant one in CSL110. Strain CSL110 was thus an appropriate subject to analyze the functional consequences of the truncation of the FtsZ N-terminal peptide. From now on we will refer to the condition of ammonium supplementation as that of ΔN-FtsZ predominance in this strain.

### Growth and Morphology of Mutant CSL110 Expressing ΔN-FtsZ

**FIGURE 7:**
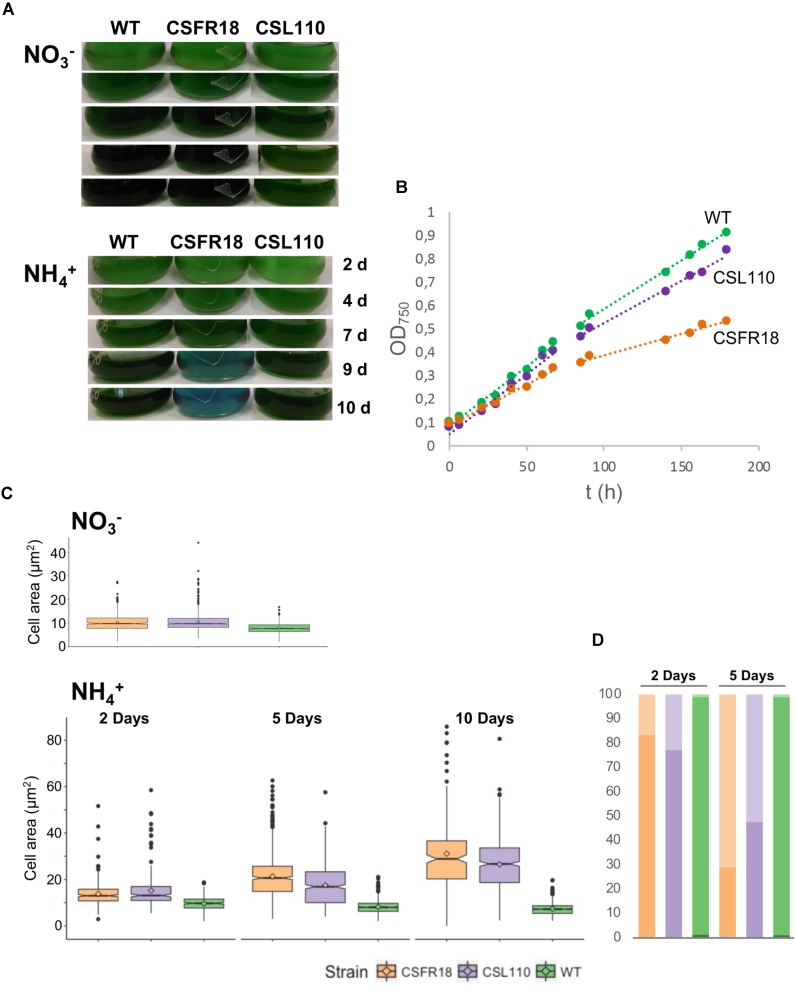
Growth and cell size effects of ΔN-FtsZ expression in a strain depleted of FtsZ. **(A)** Filaments of *Anabaena* (WT) and strains CSFR18 (P_ND_-*ftsZ*) and CSL110 (P_ND_-*ftsZ*, P*_ftsZ_*-Δ*5*′*-ftsZ*) grown in BG11 medium were collected and transferred to BG11 (${NO}_{3}^{-}$) or BG11_0_ plus ammonium (${NH}_{4}^{+}$) medium, at a cell density corresponding to 0.5 μg chlorophyll/ml, in which they were incubated under culture conditions. Cultures were photographed after the indicated times of incubation (in days). Bluish color in strain CSFR18 ammonium culture is indicative of cell lysis. **(B)** At the indicated times in BG11_0_ plus ammonium medium absorbance at 750 nm was measured. (Please, note that in strain CSL110, and specially in CSFR18, increase in cell size with regard to the WT would contribute to increase in turbidity). **(C)** Samples of the indicated cell suspensions were withdrawn, photographed under the microscope, and the obtained images (as shown in **Figure 8** below) were used for cell area determination as described in Section “Materials and Methods.” 200–300 (for all strains 2 days and CSFR18 10 days in ammonium-containing medium) or 500–600 cells (all other strains and conditions) from four different cultures were measured. Tukey’s HSD (honest significant difference) and *U* Mann–Whitney tests were performed to assess the statistical significance of comparisons. Both parameters were 0.000 for comparisons between the different strains with a given nitrogen source (meaning significant differences), except for comparison of CSFR18 and CSL110 with nitrate (0.986 and 0.337 for Tukey’s HSD and *U* Mann–Whitney tests, respectively) and 2 days with ammonium (0.574 for Tukey’s HSD) (parameters higher than 0.05, meaning non-significant differences). The mean values (diamonds in the plots) after incubation with ammonium for 10 days were: WT, 7.4 ± 2.6 μm^2^; CSFR18, 30.7 ± 15.8 μm^2^; CSL110, 26.5 ± 11.9 μm^2^. (It should be noted that the data shown for CSFR18, 10 days correspond to the persistent cells only. However, because of extensive cell lysis, these data are not representative of the culture state). **(D)** From data in **(C)**, the percentages of cells of strains CSFR18 and CSL110 with values in the 99th percentile of the WT (darker colors), and with values bigger than values in this rank (lighter colors) are represented.

**FIGURE 8:**
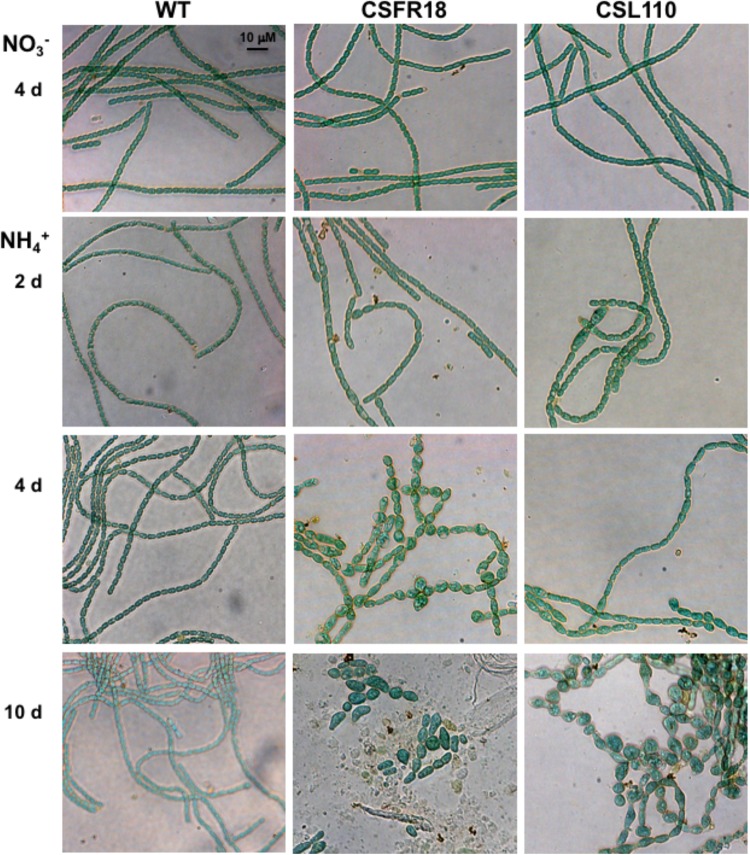
Morphological effects of ΔN-FtsZ expression in a strain depleted of FtsZ. Filaments of *Anabaena* (WT) and strains CSFR18 (P_ND_-*ftsZ*) and CSL110 (P_ND_-*ftsZ*, P*_ftsZ_*-Δ*5*′*-ftsZ*) grown in BG11 medium (${NO}_{3}^{-}$) were collected and transferred to BG11_0_ plus ammonium (${NH}_{4}^{+}$) medium, in which they were incubated under culture conditions. After the indicated times, samples of the cell suspensions were observed under a light microscope and photographed. Magnification was the same for all micrographs.

Consistent with the gene and protein expression results, both CSFR18 (Ramos-León et al., 2015) and CSL110 were able to grow in the absence of ammonium (**Figure 7A**). However, in contrast to lack of growth of CSFR18 (Ramos-León et al., 2015), strain CSL110 exhibited substantial growth in the presence of ammonium (**Figures 7A,B**). As previously described (Ramos-León et al., 2015), in medium containing nitrate the cells of strain CSFR18 appeared somewhat larger than the wild-type cells, but maintaining the cylindrical form (**Figure 8**). Under these conditions, the cells of strain CSL110 were similar to those of CSFR18 (**Figure 8**). When nitrate-grown cells were transferred to ammonium-supplemented medium (restrictive for *ftsZ* expression) strain CSFR18 formed filaments with altered morphology, including cells that first appeared elongated and later enlarged, some of them with a spherical-like shape, and which progressively were found detached from filaments and lysed (**Figure 8**; see also **Figure 7A**; Ramos-León et al., 2015). When strain CSL110 was transferred to ammonium-containing medium (conditions of native FtsZ depletion and resulting in ΔN-FtsZ predominance), it also presented conspicuous morphological alterations, including cell elongation and rounding (**Figure 8**). Detailed measurements of cellular area (**Figure 7C**) indicated that in the presence of nitrate the mean size was slightly increased to similar extents in CSFR18 and CSL110 with regard to the wild type. Upon incubation in the presence of ammonium, the average cell size in CSFR18 considerably increased with regard to the wild type, with extensive cell lysis being observed after ca. 7 days (see **Figure 7A**). Strain CSL110 also increased the average cell size, which, however, remained lower than in CSFR18 and, in contrast to CSFR18, no conspicuous cell lysis was detected in CSL110 (**Figures 7A,C**). (A representation of percentage of aberrant big cells is shown in **Figure 7D**).

### Septation Asymmetry in Strain CSL110

**FIGURE 9:**
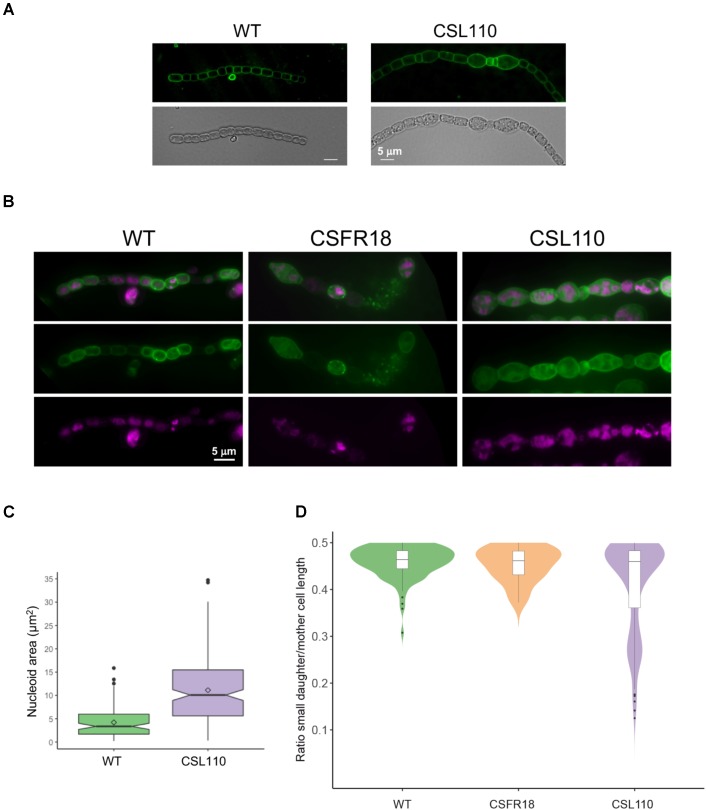
FM1-43 staining of the cytoplasmic and outer membranes and DAPI staining of nucleoids in strains CSL110, CSFR18 and *Anabaena* (WT). Filaments of the indicated strains grown in BG11 medium were incubated for 6 days **(A)**, or 5 days **(B)** under culture conditions in BG11_0_ plus ammonium medium, stained with FM1-43 (green) **(A)** or FM1-43 and DAPI (magenta) **(B)** dyes and photographed under a fluorescence microscope. In **(B)**, merged images of FM1-43 and DAPI are also shown. (Note some lysed cells in the filament of CSFR18). **(C)** DAPI images were used for nucleoid area determination as described in Section “Materials and Methods.” 80 cells for the WT and 202 for CSL110 were counted. A box plot representation of the data is shown. The mean values (diamonds in the plots) were: WT, 4.2 μm^2^; CSL110, 11.1 μm^2^. **(D)** From the FM1-43 images, cells that were dividing or had performed a division recently (identified by a long, straight septum) were visually selected, and the length of mother and daughter cells was estimated manually. The ratio of the smaller daughter to the mother (total length of both daughters) is plotted as an indication of division asymmetry. For each strain, filaments from up to four independent experiments were used and considered as a single population after assessing the similarity of their size distribution with a non-parametric Kruskal–Wallis *H* test. Total cell numbers were: 116 (WT), 162 (CSL110), 22 (CSFR18, in which due to restricted cell division few dividing cells could be detected). A combination of violin plot and box plot representations is shown for each strain.

Detailed inspection of cell morphology by fluorescence microscopy after membrane staining with dye FM1-43 (see Schneider et al., 2007) showed frequent instances of eccentric septation in strain CSL110 incubated under conditions of ΔN-FtsZ predominance (see e.g., **Figures 9A,B**). This was especially noticed in the bigger cells, and led to the apparent compartmentalization of cellular parts with different sizes. Asymmetric septation was not observed in CSFR18 or the wild type. Combined FM1-43 (membrane) and DAPI (nucleoid) staining showed that the enlarged cells of CSL110 contained DNA, frequently arranged in more than one nucleoid, and that the small cells generally contained some DNA (**Figure 9B**). Estimation of nucleoid area from images of DAPI staining showed that, following the increased cell size (**Figure 7C**), the mean nucleoid size was higher in CSL110 than in the wild type (**Figure 9C**). Under normal growth conditions, *Anabaena* possesses multiple chromosome copies, ca. 8 on average, and after cell division the ratio of the DNA content of the two daughter cells follows a Gaussian distribution, indicating some degree of randomness during partitioning (Hu et al., 2007). Polyploidy together with random chromosome partitioning between daughter cells during cell division would favor that asymmetric small cells of strain CSL110 could acquire some chromosomes.

As an estimation of the degree of cell division asymmetry, we measured the relative length of the two daughter cells resulting from a cell division event in strains CSFR18, CSL110 and wild-type *Anabaena*. **Figure 9D** shows the plot of the ratio between the lengths of the smaller daughter and the mother cells, which for a perfectly symmetric division should correspond to 0.5. Although the median value of this parameter was similar for the three strains (WT: 0.46 ± 0.03; CSFR18: 0.45 ± 0.04; CSL110: 0.41 ± 0.10), the percentile range and outlier frequency, and thus the dispersion of the values, were similar for CSFR18 and the wild type, but considerably higher for CSL110. Thus, the variability in the size of daughters, and thus of septum position, is higher in strain CSL110 than in CSFR18, which exhibits a pattern similar to the wild type.

### Localization of ΔN-FtsZ

**FIGURE 10:**
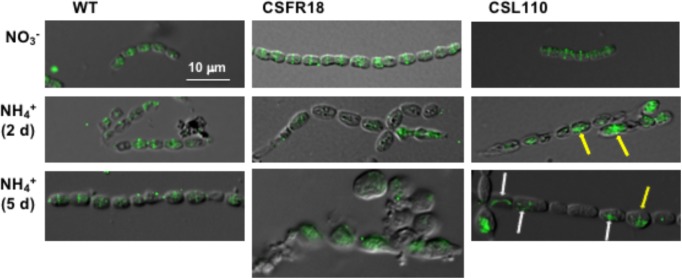
Immunofluorescence localization of FtsZ in a strain expressing ΔN-FtsZ. BG11-grown filaments of *Anabaena* (WT), CSFR18 (P_ND_-*ftsZ*) and CSL110 (P_ND_-*ftsZ*, P*_ftsZ_*-Δ*5*′*-ftsZ*) were incubated under culture conditions in BG11 (${NO}_{3}^{-}$) or BG11_0_ plus ammonium (${NH}_{4}^{+}$) medium and then prepared for immunofluorescence with anti-*Anabaena* FtsZ-protein antibodies and visualized by fluorescence microscopy. Magnification was the same for all micrographs. Arrows in ammonium-incubated CSL110 filaments point to FtsZ ring-like structures (yellow), or to aberrant FtsZ structures (white). Merged bright-field and fluorescence images are shown.

To test the formation of FtsZ rings in strain CSL110 we performed immunolocalization with antibodies raised against *Anabaena* FtsZ, which will reveal both FtsZ and ΔN-FtsZ. Filaments of strain CSL110 incubated in the presence of nitrate or ammonium (condition of ΔN-FtsZ predominance) and, for comparison, of CSFR18 and *Anabaena* were used (**Figure 10**). Under all conditions tested, *Anabaena* showed many cells with fluorescence located in a midcell ring. In contrast, strain CSL110 showed clear rings only in filaments incubated in the absence of ammonium. After transfer to ammonium-containing medium, some fluorescence in ring-like structures could be detected, either located at midcell or at eccentric positions. In addition, aberrant string-like structures were detected, and a considerable amount of the fluorescence appeared in dots or amorphous spots. Thus, ΔN-FtsZ appears impaired to form normal Z-rings at midcell. In strain CSFR18 midcell rings could be detected in the absence of ammonium. In the presence of ammonium only low and diffuse fluorescence signals could be detected.

**FIGURE 11:**
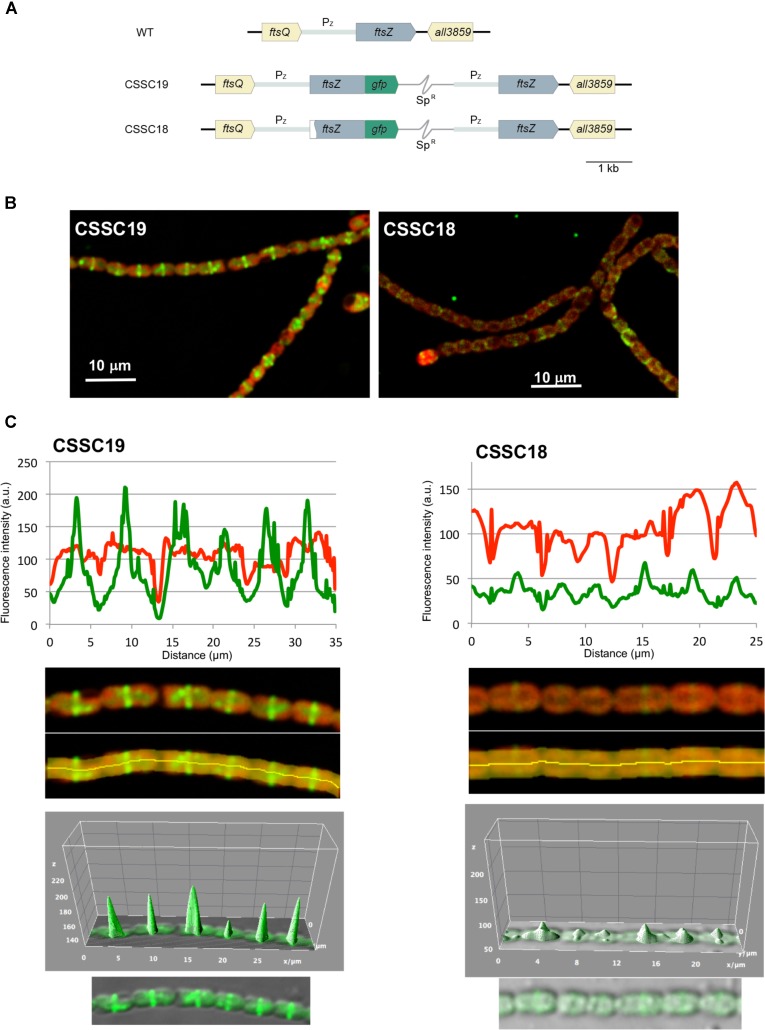
*In vivo* localization of ΔN-FtsZ-GFP protein. **(A)** Genomic structure of strains CSSC19, expressing an FtsZ protein C-terminally fused to mut2-GFP, and CSSC18, expressing ΔN-FtsZ-mut2-GFP. A white sector in the left part of a *ftsZ* ORF denotes a deletion of the 2nd to 52nd codons rendering gene Δ*5*′*-ftsZ*. Sp^R^ represents a Sp-resistance genetic determinant included in the plasmid inserted in the chromosome (thin gray trace) (see section “Materials and Methods” for details). **(B)** Filaments of strains CSSC19 and CSSC18 grown in BG11_0_ plus ammonium medium were visualized by confocal microscopy. Merged images of cyanobacterial autofluorescence (red) and GFP fluorescence (green) are shown. **(C)** GFP fluorescence (green) and autofluorescence (red) were recorded along a representative filament stretch (upper panels) of strains CSSC19 and CSSC18 in the area covered by the manually defined orange area (lower panels). Note that, whereas in both strains the autofluorescence decays in the intercellular zones, in CSSC19 the GFP fluorescence peaks at midcell. A tridimensional plot of the GFP fluorescence is shown in the lower part of the figure.

It has been reported that when an *ftsZ-gfp* gene is expressed in *Anabaena* from a heterologous copper-regulated promoter, fluorescent Z-ring-like structures are detected at midcell positions (Sakr et al., 2006). To test whether the ΔN-FtsZ protein can localize in Z-ring-like structures, *Anabaena* derivatives CSSC18 and, as a reference, CSSC19 were generated, which included a Δ*5*′-*ftsZ-mut2gfp* and an *ftsZ-mut2gfp* gene, respectively (**Figure 11A**). These strains expressed an FtsZ (strain CSSC19) or ΔN-FtsZ (strain CSSC18) protein C-terminally fused to mut2-GFP together with the native FtsZ protein. GFP fluoresce and, as reference, cyanobacterial autofluorescence was analyzed in strains CSSC19 and CSSC18 (see **Figure 11B** for representative images). Strain CSSC18 exhibited lower GFP fluorescence than CSSC19. GFP fluorescence signal versus red autofluorescence signal was ca. 0.8 for CSSC19 (counted over 88 cells) and ca. 0.5 for CSSC18 (counted over 76 cells), respectively (an example of the recording of GFP and autofluorescence along a short filament stretch is shown in the upper part of **Figure 11C**). In addition, in strain CSSC19 fluorescence was mostly observed in Z-ring-like structures located at midcell, whereas in CSSC18 fluorescence was frequently arranged in incomplete rings (note some two-peaked fluorescence in the lower part of **Figure 11C**) and, occasionally, in aberrant arrangements (see fluorescence outside the peaks in the lower part of **Figure 11C**). Thus, the ΔN-FtsZ protein appears to be impaired for the formation of Z-rings even in the presence of native FtsZ protein.

### BACTH Assays of *Anabaena* FtsZ and ΔN-FtsZ Interactions

**FIGURE 12:**
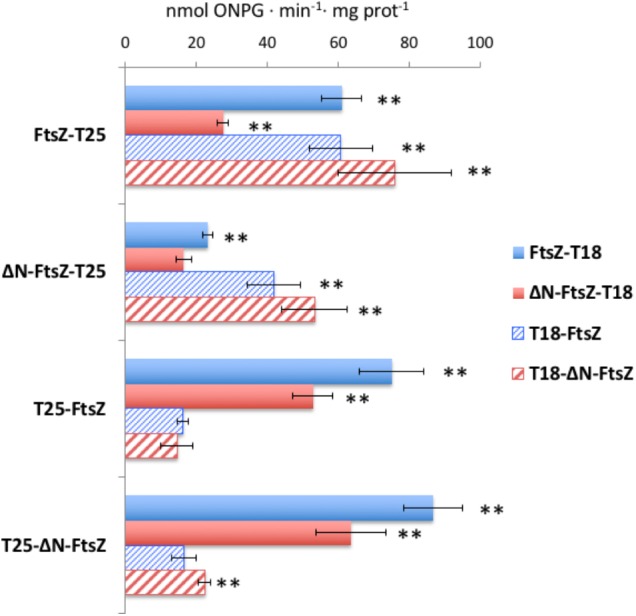
Bacterial two-hybrid analysis of FtsZ and ΔN-FtsZ interactions. Interactions of T25- and T18-fusion proteins produced in *E. coli* were measured as β-galactosidase activity in liquid cultures. The topology of the fusion is indicated by the order of components (T18-protein and T25-protein denotes the corresponding adenylate cyclase domain fused to the N-terminus of the tested protein; protein-T18 and protein-T25 denotes the corresponding adenylate cyclase domain fused to the C-terminus of the tested protein). Values (nmol ONPG⋅min^-1^⋅mg prot^-1^) are given as mean ± standard deviation. The significance of differences as assessed by Student’s *t*-tests is shown (^∗∗^*p* < 0.01; values with regard to the two respective controls: bacteria containing one of the two plasmids encoding a fused gene and the complementary empty vector) (see **Supplementary Table S2** for further details).

Bacterial two hybrid assays based on the reconstitution of adenylate cyclase from *Bordetella pertussis* were performed to test self-interactions of FtsZ or ΔN-FtsZ proteins, as well as co-interactions between the two proteins (**Figure 12** and **Supplementary Table S2**). Except in one combination of fusion proteins [T18-FtsZ/T25-FtsZ), FtsZ interacted with itself. (It is worth noting that N-terminal fusions to *E. coli* FtsZ have been described to give negative results in BACTH analysis (Karimova et al., 2005)]. Also, ΔN-FtsZ self-interaction was detected, except with the combination of fusion proteins ΔN-FtsZ-T25/ΔN-FtsZ-T18. The differences in the results of some combinations of tagged proteins involving FtsZ or ΔN-FtsZ could suggest that the geometry of self-interaction is different for the two proteins. Regarding tests between FtsZ and ΔN-FtsZ, interaction was observed with all combinations except T25-FtsZ/T18-ΔN-FtsZ and T25-ΔN-FtsZ/T18-FtsZ.

Interactions of FtsZ and ΔN-FtsZ with the *Anabaena* SepF and MinC homologs and the cyanobacterial-specific divisome factor ZipN were also tested (**Supplementary Table S2**). Regarding ZipN, only the fusion T25-ZipN could be cloned. Positive interactions were obtained with T25-ZipN and either T18-FtsZ or T18-ΔN-FtsZ, and with FtsZ-T18, but not ΔN-FtsZ-T18. Thus, both FtsZ and ΔN-FtsZ interact with ZipN, although ΔN-FtsZ could be somewhat impaired for this interaction. Regarding SepF, positive interactions were observed with the pairs T25-SepF/FtsZ-T18, SepF-T18/FtsZ-T25, and SepF-T25/FtsZ-T18, but no interaction involving ΔN-FtsZ was detected. Thus, deletion of the N-terminal peptide of FtsZ appears to impair interaction with SepF. Finally, only weak interactions could be detected involving MinC, which were more significant with ΔN-FtsZ than with FtsZ.

## Discussion

In many cyanobacteria, FtsZ includes a significant N-terminal peptide preceding the conserved globular core characteristic of FtsZ proteins, and this region is highly conserved in the clade of filamentous heterocyst-forming strains. Such conservation suggests a constraint in evolution, which might be imposed by the need to preserve a function. This is consistent with the fact that an *Anabaena* derivative producing only a version of FtsZ lacking the distinct N-terminal peptide expressed from the native *ftsZ* gene promoter could not be segregated, indicating that this version of the protein is unable to provide all the FtsZ functions required for viability.

According to BACTH and *in vitro* polymerization assays, both FtsZ and ΔN-FtsZ are able of self-interactions to form polymers. FtsZ from *E. coli* polymerizes *in vitro* adopting a variety of conformations including linear (protofilaments, sheets and bundles) and curved arrays, which are strongly influenced by the assay conditions. Indeed, this is a dynamic process influenced by GTP-binding and hydrolysis, in such a way that GTP-binding promotes polymerization and, after hydrolysis and Pi release, GDP-bound polymers have more tendency to curve and disassemble. Thus, when incubated in diluted buffers in the presence of GTP, the single straight GTP-bound protofilament appears to represent the predominant form, which prevails as long as GTP is present (Mukherjee and Lutkenhaus, 1998; Lu et al., 2000; Romberg and Mitchinson, 2004; see Romberg and Levin, 2003; Erickson et al., 2010). In contrast, when incubated in diluted buffers with GTP filaments of *Anabaena* FtsZ appear flexible and curly forming aggregates of structures similar to the toroids formed by *E. coli* FtsZ in the presence of crowding agents (Popp et al., 2009). It would be possible that the extensively curled filaments of *Anabaena* FtsZ that we have observed corresponded to GDP-bound polymers. However, given the level of GTPase activity of this enzyme, the concentration of GTP remaining at the onset of sample preparation for TEM would still be ca. 2 mM, which would disfavor this possibility.

In the tertiary structure of *M. jannaschii* FtsZ, an N-terminal peptide of ca. 33-amino acids shows sticking out of the GTPase domain (Löwe and Amos, 1998). In this region, residues 1–20 appear to be disordered (Löwe and Amos, 1998; PDB entry 1W5A) or forming a helix (H-1) in the dimeric structure of the protein (Oliva et al., 2004) and residues 24–34 conform an α-helix [H0 (H2 in **Figure 1B**)] (see **Figure 1**). Both H-1 and H0 are located at the filament surface, with H0, which is not present in *E. coli*, being the only longitudinal contact between two consecutive N-terminal domains (Oliva et al., 2004). It is possible that the longer (ca. 60-residues) N-terminal peptide of *Anabaena* FtsZ participates in longitudinal contacts at the filament surface bridging consecutive FtsZ subunits, which could promote curling in the filaments.

In contrast to the toroid-like structures formed by *Anabaena* FtsZ, ΔN-FtsZ form thick bundles of straight filaments. It is worth noting that because the detected GTPase activities of FtsZ and ΔN-FtsZ are not very different (activity of ΔN-FtsZ is ca. 0.6 that of FtsZ), it is unlikely that the large difference in bundling between the two proteins results from differences in the bound nucleotide (GTP versus GDP) as a result of the rate of GTP hydrolysis. The observed thick arrays of ΔN-FtsZ closely resemble those of *E. coli* FtsZ assembled in the presence of factors such as CaCl_2_ (Lu et al., 2000) or ZipA (Hale et al., 2000) or of *B. subtilis* FtsZ in the presence of ZapA (Gueiros-Filho and Losick, 2002) or SepF, at pH 6.8 (Singh et al., 2008), all of which increase the assembly and bundling of FtsZ polymers. In the case of protein factors, they have been proposed to induce FtsZ bundling by stabilizing lateral interactions between FtsZ protofilaments. Thus, regarding the *Anabaena* proteins, lateral interactions with ΔN-FtsZ appear increased in comparison to FtsZ. Perhaps the N-terminal peptide of *Anabaena* FtsZ is important to maintain a suitable distance for lateral interactions between FtsZ units.

To study the *in vivo* effects of deleting the N-terminal peptide of *Anabaena* FtsZ, we generated strain CSL110, which expresses both FtsZ and ΔN-FtsZ in different proportions depending on the incubation conditions. When filaments of CSL110 are transferred to medium with ammonium, FtsZ is progressively depleted so that ΔN-FtsZ becomes the preponderant form. Under these conditions the average cell size progressively increases, in comparison to the wild type, in both CSL110 and its parental strain CSFR18, which presents only depleted FtsZ. However, the average cell size remains lower in CSL110 than in CSFR18, in which size increase results in extensive cell lysis, not observed in CSL110. Cell size increase reflects impairment in cell division.

In strain CSFR18 the frequency of cell division likely reflects the amount of FtsZ present in the cells, which becomes negligible after prolonged incubation in the presence of ammonium. In strain CSL110, division frequency could reflect the total amount of protein (FtsZ + ΔN-FtsZ). Although in this strain the Δ*5*′*-ftsZ* allele is expressed from the native P*_ftsZ_* promoter, the levels of ΔN-FtsZ are also observed to decrease upon incubation with ammonium, becoming lower than those of FtsZ in the wild type (**Figure 6D**, see 5 days). Decreased stability of ΔN-FtsZ as compared to FtsZ could explain this effect. In this scenario, the low stability of ΔN-FtsZ *in vivo* would be a consequence of its lacking of the N-terminal peptide, and could be related to the different mode of *in vitro* polymerization as compared to FtsZ. Consistently, the expression levels of ΔN-FtsZ-GFP (in strain CSSC18) are lower than of FtsZ-GFP (in strain CSSC19).

Furthermore, in strain CSL110 the ΔN-FtsZ cellular levels are lower when the amount of FtsZ is already very low (after 5 days in the presence of ammonium), and higher at shorter times in the presence of ammonium -2 days- or in the presence of nitrate. Thus, the interaction between FtsZ and ΔN-FtsZ *in vivo* could protect to some extent the latter from degradation, so that instability of ΔN-FtsZ would be higher in the absence of FtsZ than in its presence. Indeed, our observations of ΔN-FtsZ-GFP localization in strain CSSC18, as well as BACTH results, suggest both that ΔN-FtsZ can interact with wild-type FtsZ and that the mixed polymers behaves differently than those formed only by FtsZ.

Besides impairment in the extent of cell division, instances of eccentric septation and size asymmetry between daughter cells are observed in CSL110 but not in CSFR18 or the wild type (**Figures 9A,B,D**). These comparisons suggest that asymmetric cell division in CSL110 results from the expression of ΔN-FtsZ, and thus that the N-terminal peptide of *Anabaena* FtsZ is important for efficient localization of the correct division plane. Also, the maintenance of viable large cells in CSL110, as a result of partial compensation of FtsZ depletion by expression of ΔN-FtsZ, could permit the observation of asymmetric septation in those large cells.

Our BACTH assays involving the common Z-ring positioner MinC factor (**Supplementary Table S2**) suggest that its interaction with ΔN-FtsZ could be altered with regard to MinC/FtsZ interaction. This impairment could contribute to alter the spatial regulation of the Z-ring in strain CSL110. However, given that with all combinations of fusion proteins tested the detected interactions were weak, the action of MinC should be further investigated. In addition, our BACTH results (**Supplementary Table S2**) suggest that, in contrast to FtsZ, ΔN-FtsZ is impaired for interaction with SepF, an effect that could result from altered lateral interactions between ΔN-FtsZ protofilaments. Whatever the mechanism, impaired interactions of polymers formed by ΔN-FtsZ, or mixed FtsZ/ΔN-FtsZ polymers, with SepF, which according to our unpublished results is an essential protein in *Anabaena*, could represent a main factor impairing cell division in strain CSL110.

In summary, the N-terminal peptide of *Anabaena* FtsZ appears to be essential for the correct longitudinal and lateral FtsZ interactions that are needed to establish a functional FtsZ ring, as well as for FtsZ interaction with at least one essential partner -SepF-, with an effect in the extent of cell division and division plane location and, hence, in cell viability. The distinct features of cell division in *Anabaena* underscore the importance of studying key bacterial processes in a wide range of bacterial types.

## Author Contributions

LC-G, SC, AV, SP, JO, and AH designed the research. LC-G, SC, AV, SP, and JO performed the research. LC-G, SC, AV, SP, JO, IL, and AH analyzed the data. AH directed research. AH and JO wrote the paper.

## Conflict of Interest Statement

The authors declare that the research was conducted in the absence of any commercial or financial relationships that could be construed as a potential conflict of interest.

## Footnotes

1 http://imagej.nih.gov/ij/

2 https://www.rstudio.com/products/rstudio/
