# Supplementary material for: FtsZ of Filamentous, Heterocyst-Forming Cyanobacteria Has a Conserved N-Terminal Peptide Required for Normal FtsZ Polymerization and Cell Division
Source: Front Microbiol. 2018 Oct 2;9:2260. doi: 10.3389/fmicb.2018.02260 (PMC6175996; doi:10.3389/fmicb.2018.02260)
Supplement: Supplementary file 1 [file Data_Sheet_1.PDF]

Fig. S1

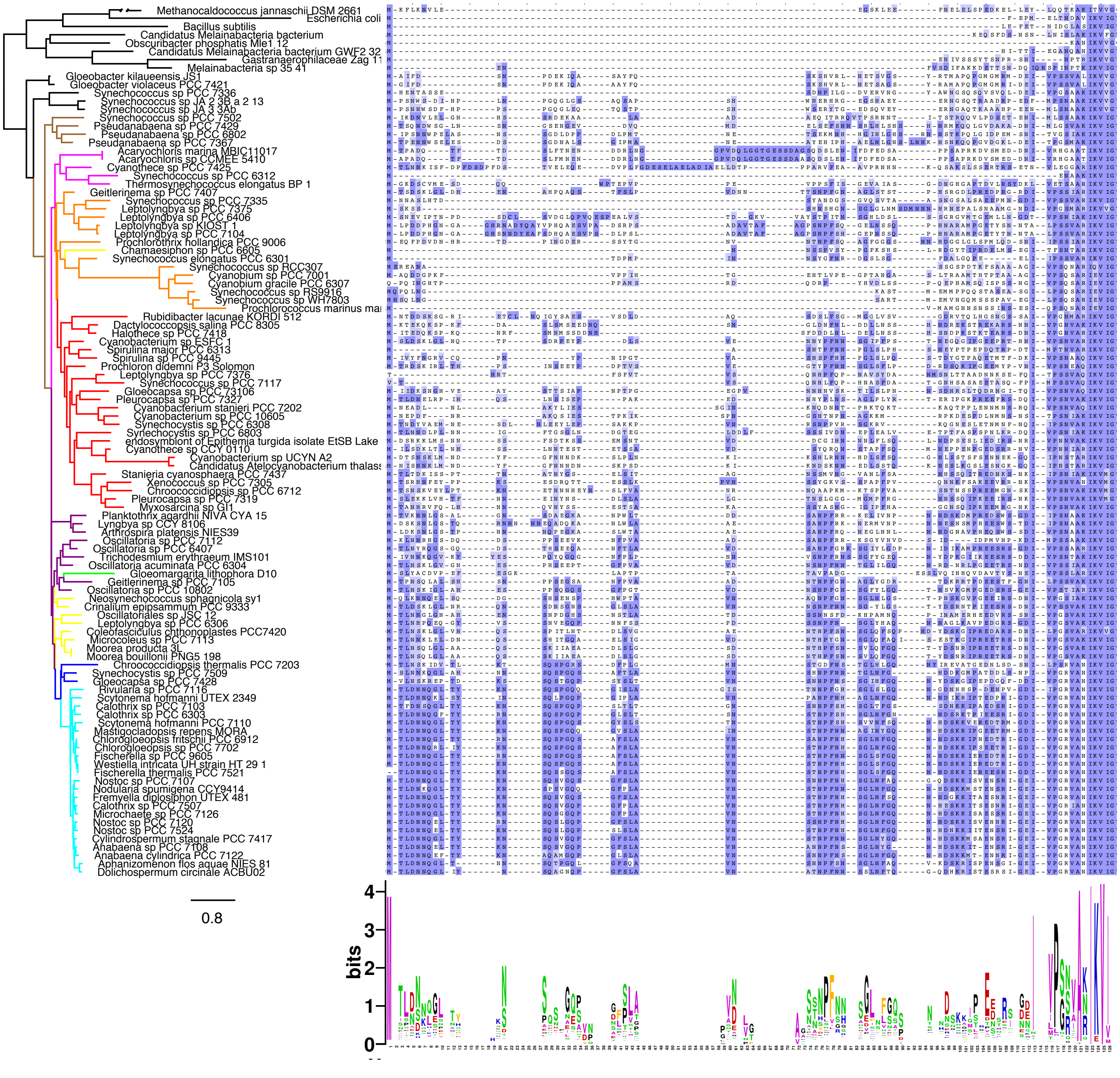
Methanocaldococcus jannaschii DSM 2661
Escherichia coli
Bacillus subtilis
Candidatus Melainabacteria bacterium
Obscuribacter phosphatis Mle1 12
Candidatus Melainabacteria bacterium GWF2 32
Gastranaerophilaceae Zag 11
Melainabacteria sp 35 41
Gloeobacter kilaueensis JS1
Gloeobacter violaceus PCC 7421
Synechococcus sp PCC 7336
Synechococcus sp JA 2 3B a 2 13
Synechococcus sp JA 3 3Ab
Synechococcus sp PCC 7502
Pseudanabaena sp PCC 7429
Pseudanabaena sp PCC 6802
Pseudanabaena sp PCC 7367
Acaryochloris marina MBIC11017
Acaryochloris sp CCMEE 5410
Cyanothece sp PCC 7425
Synechococcus sp PCC 6312
Thermosynechococcus elongatus BP 1
Geitlerinema sp PCC 7407
Synechococcus sp PCC 7335
Leptolyngbya sp PCC 7375
Leptolyngbya sp PCC 6406
Leptolyngbya sp KIOST 1
Leptolyngbya sp PCC 7104
Prochlorothrix hollandica PCC 9006
Chamaesiphon sp PCC 6605
Synechococcus elongatus PCC 6301
Synechococcus sp RCC307
Cyanobium sp PCC 7001
Cyanobium gracile PCC 6307
Synechococcus sp RS9916
Synechococcus sp WH7803
Prochlorococcus marinus mar
Rubidibacter lacunae KORDI 51 2
Dactylococcopsis salina PCC 8305
Halothece sp PCC 7418
Cyanobacterium sp ESFC 1
Spirulina major PCC 6313
Spirulina sp PCC 9445
Prochloron didemni P3 Solomon
Leptolyngbya sp PCC 7376
Synechococcus sp PCC 7117
Gloeocapsa sp PCC 73106
Pleurocapsa sp PCC 7327
Cyanobacterium stanieri PCC 7202
Cyanobacterium sp PCC 10605
Synechocystis sp PCC 6308
Synechocystis sp PCC 6803
endosymbiont of Epithemia turgida isolate EtSB Lake
Cyanothece sp CCY 0110
Cyanobacterium sp UCYN A2
Candidatus Atelocyanobacterium thalass
Stanieria cyanosphaera PCC 7437
Xenococcus sp PCC 7305
Chroococcidiopsis sp PCC 6712
Pleurocapsa sp PCC 7319
Myxosarcina sp GI1
Planktothrix agardhii NIVA CYA 15
Lyngbya sp CCY 8106
Arthrospira platensis NIES39
Oscillatoria sp PCC 7112
Oscillatoria sp PCC 6407
Trichodesmium erythraeum IMS101
Oscillatoria acuminata PCC 6304
Gloeomargarita lithophora D10
Geitlerinema sp PCC 7105
Oscillatoria sp PCC 10802
Neosynechococcus sphagnicola sy1
Crinalium epipsammum PCC 9333
Oscillatoriales sp JSC 12
Leptolyngbya sp PCC 6306
Coleofasciculus chthonoplastes PCC7420
Microcoleus sp PCC 7113
Moorea producta 3L
Moorea bouillonii PNG5 198
Chroococcidiopsis thermalis PCC 7203
Synechocystis sp PCC 7509
Gloeocapsa sp PCC 7428
Rivularia sp PCC 7116
Scytonema hofmanni UTEX 2349
Calothrix sp PCC 7103
Calothrix sp PCC 6303
Scytonema hofmanni PCC 7110
Mastigocladopsis repens MORA
Chlorogloeopsis fritschii PCC 6912
Chlorogloeopsis sp PCC 7702
Fischerella sp PCC 9605
Westiella intricata UH strain HT 29 1
Fischerella thermalis PCC 7521
Nostoc sp PCC 7107
Nodularia spumigena CCY9414
Fremyella diplosiphon UTEX 481
Calothrix sp PCC 7507
Microchaete sp PCC 7126
Nostoc sp PCC 7120
Nostoc sp PCC 7524
Cylindrospermum stagnale PCC 7417
Anabaena sp PCC 7108
Anabaena cylindrica PCC 7122
Aphanizomenon flos aquae NIES 81
Dolichospermum circinale ACBU02
0.8
bits
4
3
2
1
0

Fig. S2

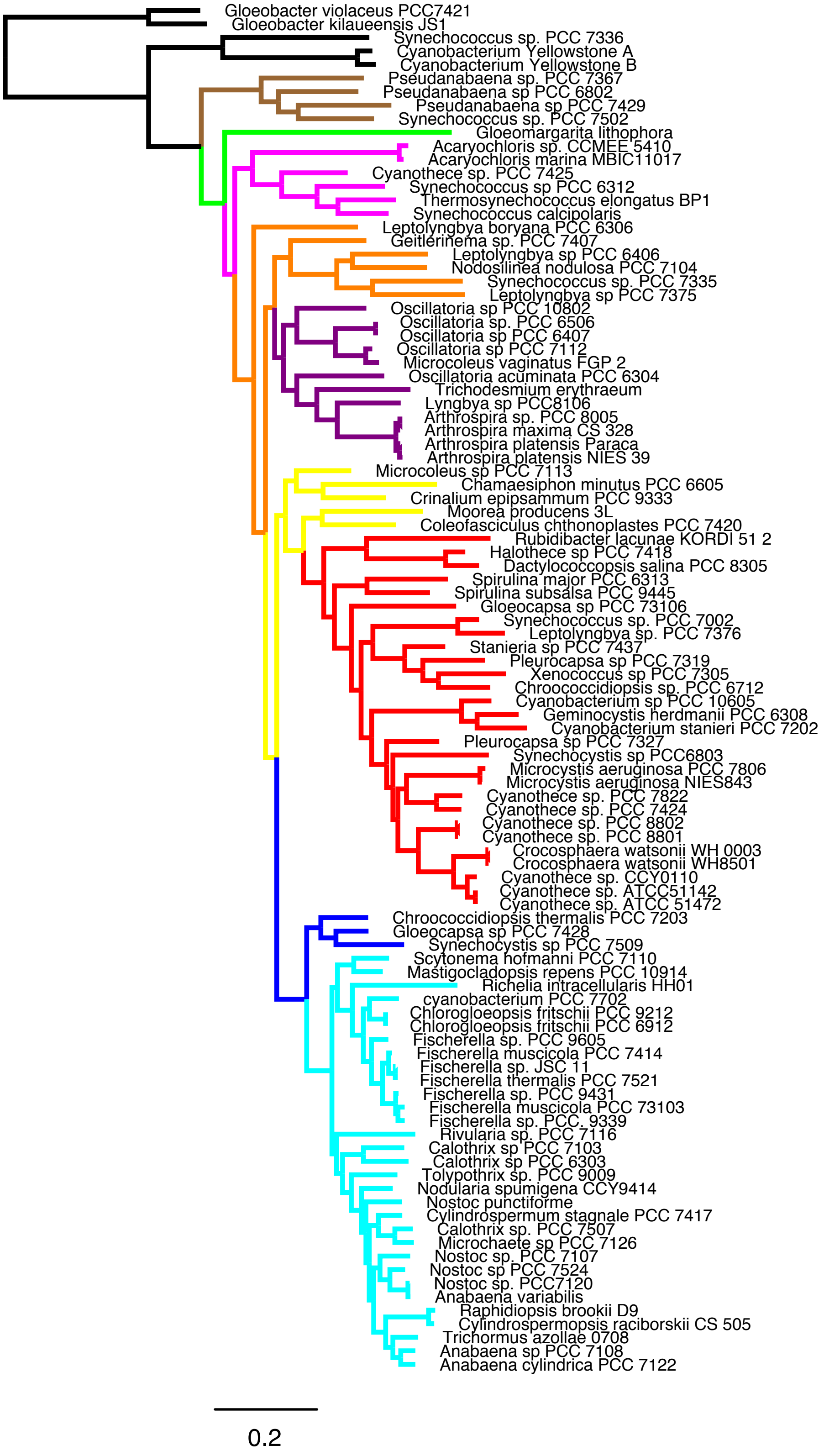

Gloeobacter violaceus PCC7421
Gloeobacter kilaueensis JS1
Synechococcus sp. PCC 7336
Cyanobacterium Yellowstone A
Cyanobacterium Yellowstone B
Pseudanabaena sp. PCC 7367
Pseudanabaena sp PCC 6802
Pseudanabaena sp PCC 7429
Synechococcus sp. PCC 7502
Gloeomargarita lithophora
Acaryochloris sp. CCMEE 5410
Acaryochloris marina MBIC11017
Cyanothece sp. PCC 7425
Synechococcus sp PCC 6312
Thermosynechococcus elongatus BP1
Synechococcus calcipolaris
Leptolyngbya boryana PCC 6306
Geitlerinema sp. PCC 7407
Leptolyngbya sp PCC 6406
Nodosilinea nodulosa PCC 7104
Synechococcus sp. PCC 7335
Leptolyngbya sp PCC 7375
Oscillatoria sp PCC 10802
Oscillatoria sp. PCC 6506
Oscillatoria sp PCC 6407
Oscillatoria sp PCC 7112
Microcoleus vaginatus FGP 2
Oscillatoria acuminata PCC 6304
Trichodesmium erythraeum
Lyngbya sp PCC8106
Arthrospira sp. PCC 8005
Arthrospira maxima CS 328
Arthrospira platensis Paraca
Arthrospira platensis NIES 39
Microcoleus sp PCC 7113
Chamaesiphon minutus PCC 6605
Crinalium epipsammum PCC 9333
Moorea producens 3L
Coleofasciculus chthonoplastes PCC 7420
Rubidibacter lacunae KORDI 51 2
Halothece sp PCC 7418
Dactylococcopsis salina PCC 8305
Spirulina major PCC 6313
Spirulina subsalsa PCC 9445
Gloeocapsa sp PCC 73106
Synechococcus sp. PCC 7002
Leptolyngbya sp. PCC 7376
Stanieria sp PCC 7437
Pleurocapsa sp PCC 7319
Xenococcus sp PCC 7305
Chroococcidiopsis sp. PCC 6712
Cyanobacterium sp PCC 10605
Geminocystis herdmanii PCC 6308
Cyanobacterium stanieri PCC 7202
Pleurocapsa sp PCC 7327
Synechocystis sp PCC6803
Microcystis aeruginosa PCC 7806
Microcystis aeruginosa NIES843
Cyanothece sp. PCC 7822
Cyanothece sp. PCC 7424
Cyanothece sp. PCC 8802
Cyanothece sp. PCC 8801
Crocosphaera watsonii WH 0003
Crocosphaera watsonii WH8501
Cyanothece sp. CCY0110
Cyanothece sp. ATCC51142
Cyanothece sp. ATCC 51472
Chroococcidiopsis thermalis PCC 7203
Gloeocapsa sp PCC 7428
Synechocystis sp PCC 7509
Scytonema hofmanni PCC 7110
Mastigocladopsis repens PCC 10914
Richelia intracellularis HH01
cyanobacterium PCC 7702
Chlorogloeopsis fritschii PCC 9212
Chlorogloeopsis fritschii PCC 6912
Fischerella sp. PCC 9605
Fischerella muscicola PCC 7414
Fischerella sp. JSC 11
Fischerella thermalis PCC 7521
Fischerella sp. PCC 9431
Fischerella muscicola PCC 73103
Fischerella sp. PCC. 9339
Rivularia sp. PCC 7116
Calothrix sp PCC 7103
Calothrix sp PCC 6303
Tolypothrix sp. PCC 9009
Nodularia spumigena CCY9414
Nostoc punctiforme
Cylindrospermum stagnale PCC 7417
Calothrix sp. PCC 7507
Microchaete sp PCC 7126
Nostoc sp. PCC 7107
Nostoc sp PCC 7524
Nostoc sp. PCC7120
Anabaena variabilis
Raphidiopsis brookii D9
Cylindrospermopsis raciborskii CS 505
Trichormus azollae 0708
Anabaena sp PCC 7108
Anabaena cylindrica PCC 7122
0.2

Fig. S3

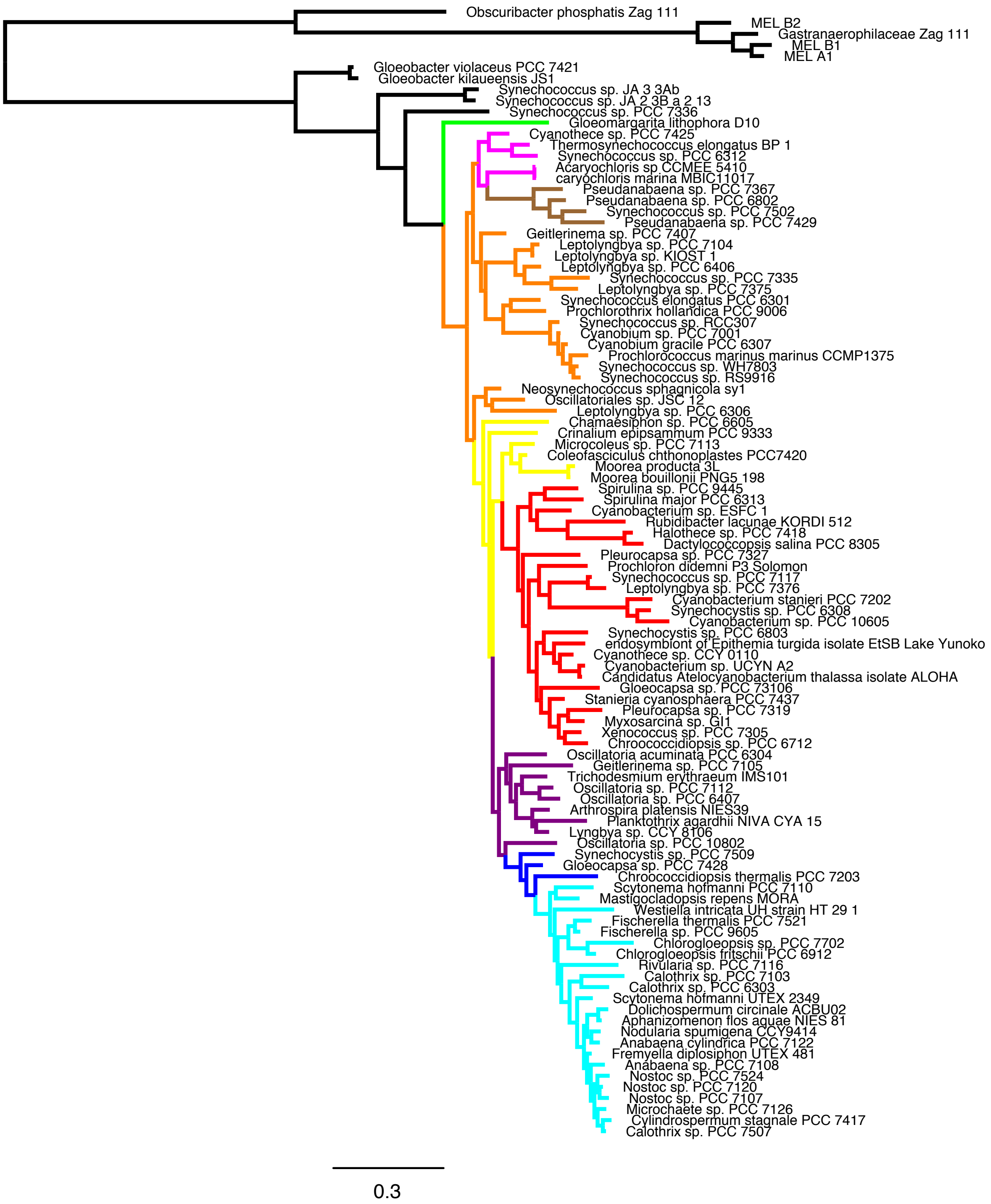

Obscuribacter phosphatis Zag 111
MEL B2
Gastranaerophilaceae Zag 111
MEL B1
MEL A1
Gloeobacter violaceus PCC 7421
Gloeobacter kilaueensis JS1
Synechococcus sp. JA 3 3Ab
Synechococcus sp. JA 2 3B a 2 13
Synechococcus sp. PCC 7336
Gloeomargarita lithophora D10
Cyanothece sp. PCC 7425
Thermosynechococcus elongatus BP 1
Synechococcus sp. PCC 6312
Acaryochloris sp CCMEE 5410
caryochloris marina MBIC11017
Pseudanabaena sp. PCC 7367
Pseudanabaena sp. PCC 6802
Synechococcus sp. PCC 7502
Pseudanabaena sp. PCC 7429
Geitlerinema sp. PCC 7407
Leptolyngbya sp. PCC 7104
Leptolyngbya sp. KIOST 1
Leptolyngbya sp. PCC 6406
Synechococcus sp. PCC 7335
Leptolyngbya sp. PCC 7375
Synechococcus elongatus PCC 6301
Prochlorothrix hollandica PCC 9006
Synechococcus sp. RCC307
Cyanobium sp. PCC 7001
Cyanobium gracile PCC 6307
Prochlorococcus marinus marinus CCMP1375
Synechococcus sp. WH7803
Synechococcus sp. RS9916
Neosynechococcus sphagnicola sy1
Oscillatoriales sp. JSC 12
Leptolyngbya sp. PCC 6306
Chamaesiphon sp. PCC 6605
Crinalium epipsammum PCC 9333
Microcoleus sp. PCC 7113
Coleofasciculus chthonoplastes PCC7420
Moorea producta 3L
Moorea bouillonii PNG5 198
Spirulina sp. PCC 9445
Spirulina major PCC 6313
Cyanobacterium sp. ESFC 1
Rubidibacter lacunae KORDI 512
Halothece sp. PCC 7418
Dactylococcopsis salina PCC 8305
Pleurocapsa sp. PCC 7327
Prochloron didemni P3 Solomon
Synechococcus sp. PCC 7117
Leptolyngbya sp. PCC 7376
Cyanobacterium stanieri PCC 7202
Synechocystis sp. PCC 6308
Cyanobacterium sp. PCC 10605
Synechocystis sp. PCC 6803
endosymbiont of Epithemia turgida isolate EtSB Lake Yunoko
Cyanothece sp. CCY 0110
Cyanobacterium sp. UCYN A2
Candidatus Atelocyanobacterium thalassa isolate ALOHA
Gloeocapsa sp. PCC 73106
Stanieria cyanosphaera PCC 7437
Pleurocapsa sp. PCC 7319
Myxosarcina sp. GI1
Xenococcus sp. PCC 7305
Chroococcidiopsis sp. PCC 6712
Oscillatoria acuminata PCC 6304
Geitlerinema sp. PCC 7105
Trichodesmium erythraeum IMS101
Oscillatoria sp. PCC 7112
Oscillatoria sp. PCC 6407
Arthrospira platensis NIES39
Planktothrix agardhii NIVA CYA 15
Lyngbya sp. CCY 8106
Oscillatoria sp. PCC 10802
Synechocystis sp. PCC 7509
Gloeocapsa sp. PCC 7428
Chroococcidiopsis thermalis PCC 7203
Scytonema hofmanni PCC 7110
Mastigocladopsis repens MORA
Westiella intricata UH strain HT 29 1
Fischerella thermalis PCC 7521
Fischerella sp. PCC 9605
Chlorogloeopsis sp. PCC 7702
Chlorogloeopsis fritschii PCC 6912
Rivularia sp. PCC 7116
Calothrix sp. PCC 7103
Calothrix sp. PCC 6303
Scytonema hofmanni UTEX 2349
Dolichospermum circinale ACBU02
Aphanizomenon flos aquae NIES 81
Nodularia spumigena CCY9414
Anabaena cylindrica PCC 7122
Fremyella diplosiphon UTEX 481
Anabaena sp. PCC 7108
Nostoc sp. PCC 7524
Nostoc sp. PCC 7120
Nostoc sp. PCC 7107
Microchaete sp. PCC 7126
Cylindrospermum stagnale PCC 7417
Calothrix sp. PCC 7507
0.3

Fig. S4

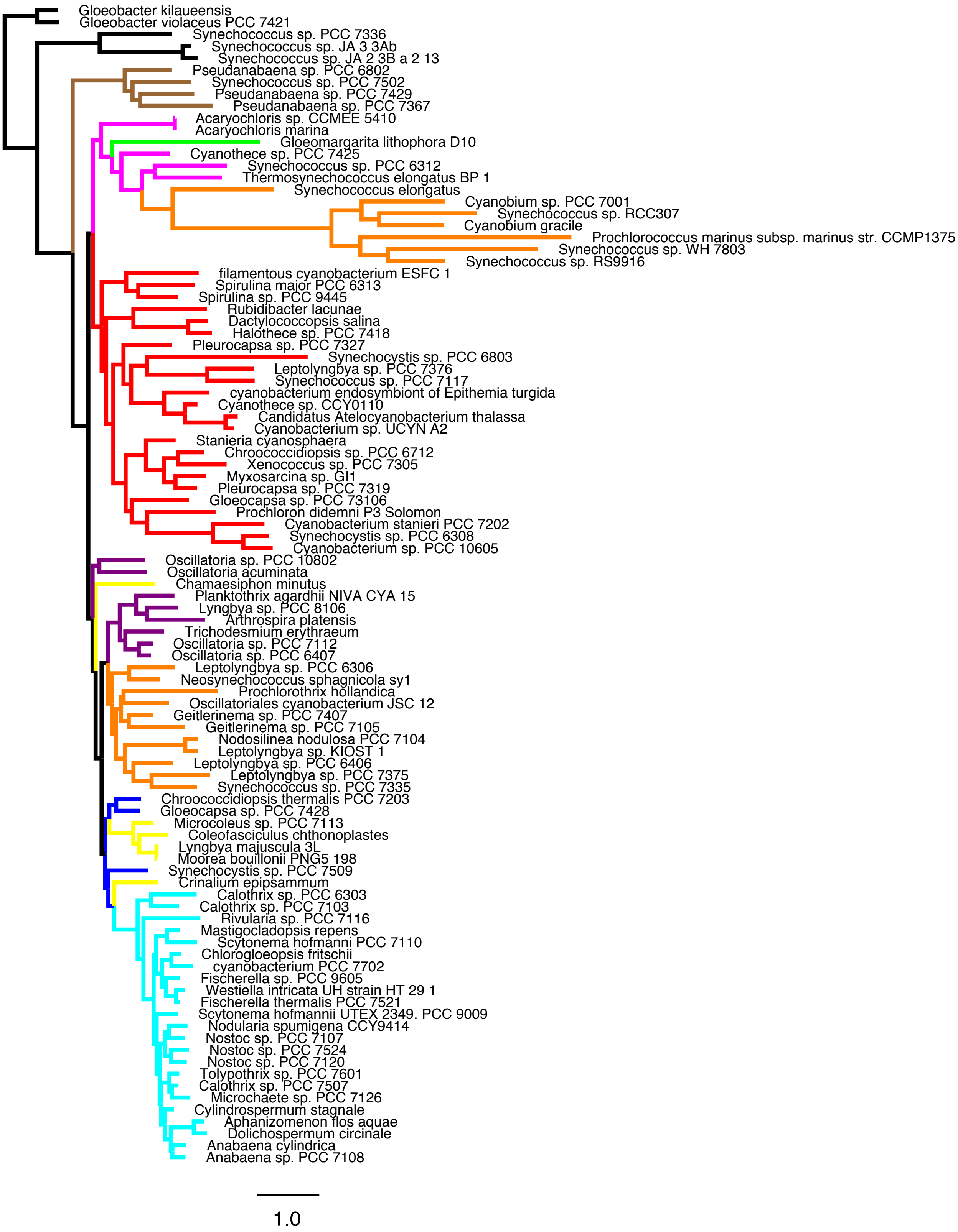

Gloeobacter kilaueensis
Gloeobacter violaceus PCC 7421
Synechococcus sp. PCC 7336
Synechococcus sp. JA 3 3Ab
Synechococcus sp. JA 2 3B a 2 13
Pseudanabaena sp. PCC 6802
Synechococcus sp. PCC 7502
Pseudanabaena sp. PCC 7429
Pseudanabaena sp. PCC 7367
Acaryochloris sp. CCMEE 5410
Acaryochloris marina
Gloeomargarita lithophora D10
Cyanothece sp. PCC 7425
Synechococcus sp. PCC 6312
Thermosynechococcus elongatus BP 1
Synechococcus elongatus
Cyanobium sp. PCC 7001
Synechococcus sp. RCC307
Cyanobium gracile
Prochlorococcus marinus subsp. marinus str. CCMP1375
Synechococcus sp. WH 7803
Synechococcus sp. RS9916
filamentous cyanobacterium ESFC 1
Spirulina major PCC 6313
Spirulina sp. PCC 9445
Rubidibacter lacunae
Dactylococcopsis salina
Halothece sp. PCC 7418
Pleurocapsa sp. PCC 7327
Synechocystis sp. PCC 6803
Leptolyngbya sp. PCC 7376
Synechococcus sp. PCC 7117
cyanobacterium endosymbiont of Epithemia turgida
Cyanothece sp. CCY0110
Candidatus Atelocyanobacterium thalassa
Cyanobacterium sp. UCYN A2
Stanieria cyanosphaera
Chroococcidiopsis sp. PCC 6712
Xenococcus sp. PCC 7305
Myxosarcina sp. GI1
Pleurocapsa sp. PCC 7319
Gloeocapsa sp. PCC 73106
Prochloron didemni P3 Solomon
Cyanobacterium stanieri PCC 7202
Synechocystis sp. PCC 6308
Cyanobacterium sp. PCC 10605
Oscillatoria sp. PCC 10802
Oscillatoria acuminata
Chamaesiphon minutus
Planktothrix agardhii NIVA CYA 15
Lyngbya sp. PCC 8106
Arthrospira platensis
Trichodesmium erythraeum
Oscillatoria sp. PCC 7112
Oscillatoria sp. PCC 6407
Leptolyngbya sp. PCC 6306
Neosynechococcus sphagnicola sy1
Prochlorothrix hollandica
Oscillatoriales cyanobacterium JSC 12
Geitlerinema sp. PCC 7407
Geitlerinema sp. PCC 7105
Nodosilinea nodulosa PCC 7104
Leptolyngbya sp. KIOST 1
Leptolyngbya sp. PCC 6406
Leptolyngbya sp. PCC 7375
Synechococcus sp. PCC 7335
Chroococcidiopsis thermalis PCC 7203
Gloeocapsa sp. PCC 7428
Microcoleus sp. PCC 7113
Coleofasciculus chthonoplastes
Lyngbya majuscula 3L
Moorea bouillonii PNG5 198
Synechocystis sp. PCC 7509
Crinalium epipsammum
Calothrix sp. PCC 6303
Calothrix sp. PCC 7103
Rivularia sp. PCC 7116
Mastigocladopsis repens
Scytonema hofmanni PCC 7110
Chlorogloeopsis fritschii
cyanobacterium PCC 7702
Fischerella sp. PCC 9605
Westiella intricata UH strain HT 29 1
Fischerella thermalis PCC 7521
Scytonema hofmannii UTEX 2349. PCC 9009
Nodularia spumigena CCY9414
Nostoc sp. PCC 7107
Nostoc sp. PCC 7524
Nostoc sp. PCC 7120
Tolypothrix sp. PCC 7601
Calothrix sp. PCC 7507
Microchaete sp. PCC 7126
Cylindrospermum stagnale
Aphanizomenon flos aquae
Dolichospermum circinale
Anabaena cylindrica
Anabaena sp. PCC 7108
1.0

Fig. S5

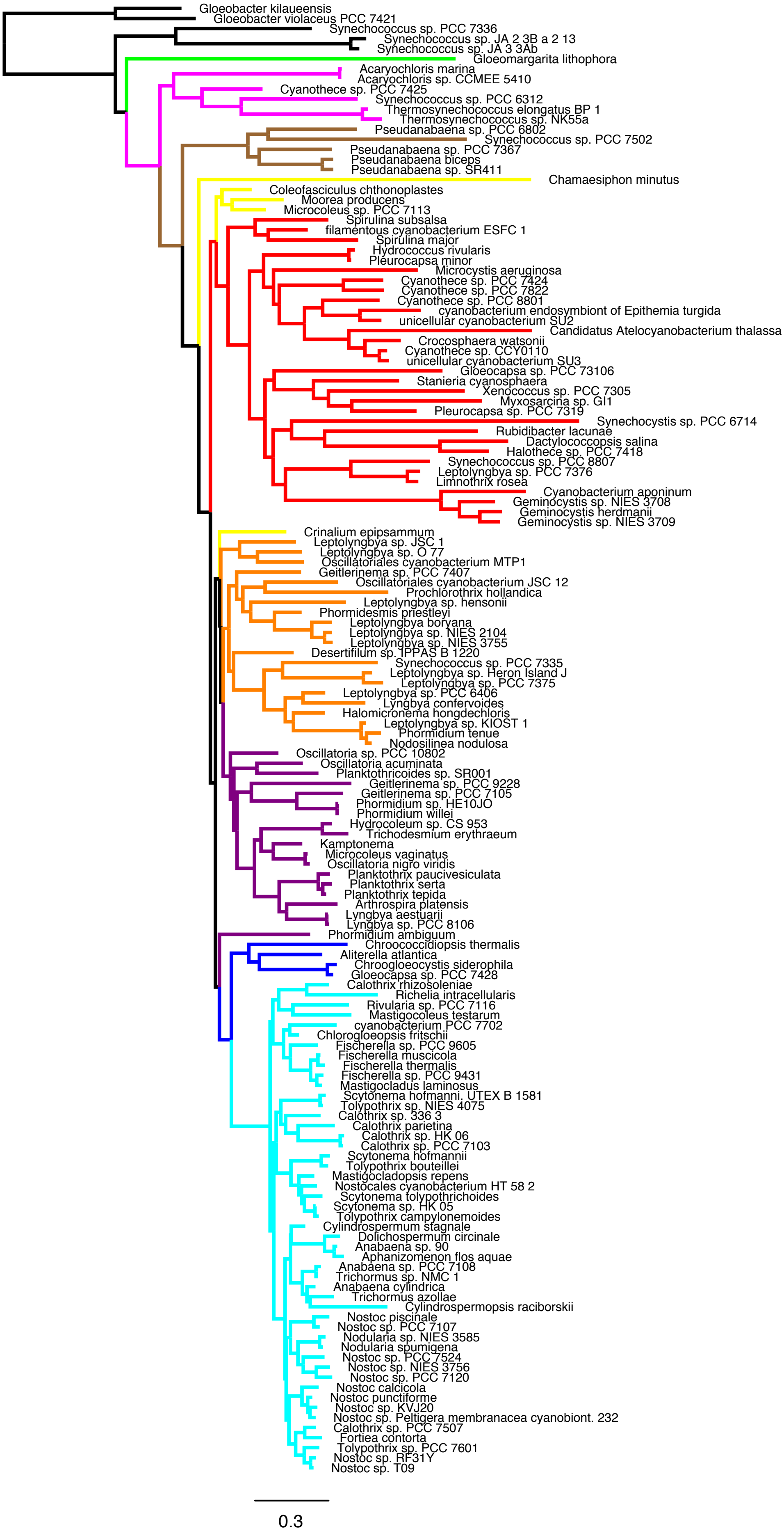
Gloeobacter kilaueensis
Gloeobacter violaceus PCC 7421
Synechococcus sp. PCC 7336
Synechococcus sp. JA 2 3B a 2 13
Synechococcus sp. JA 3 3Ab
Gloeomargarita lithophora
Acaryochloris marina
Acaryochloris sp. CCMEE 5410
Cyanothece sp. PCC 7425
Synechococcus sp. PCC 6312
Thermosynechococcus elongatus BP 1
Thermosynechococcus sp. NK55a
Pseudanabaena sp. PCC 6802
Synechococcus sp. PCC 7502
Pseudanabaena sp. PCC 7367
Pseudanabaena biceps
Pseudanabaena sp. SR411
Chamaesiphon minutus
Coleofasciculus chthonoplastes
Moorea producens
Microcoleus sp. PCC 7113
Spirulina subsalsa
filamentous cyanobacterium ESFC 1
Spirulina major
Hydrococcus rivularis
Pleurocapsa minor
Microcystis aeruginosa
Cyanothece sp. PCC 7424
Cyanothece sp. PCC 7822
Cyanothece sp. PCC 8801
cyanobacterium endosymbiont of Epithemia turgida
unicellular cyanobacterium SU2
Candidatus Atelocyanobacterium thalassa
Crocosphaera watsonii
Cyanothece sp. CCY0110
unicellular cyanobacterium SU3
Gloeocapsa sp. PCC 73106
Stanieria cyanosphaera
Xenococcus sp. PCC 7305
Myxosarcina sp. GI1
Pleurocapsa sp. PCC 7319
Synechocystis sp. PCC 6714
Rubidibacter lacunae
Dactylococcopsis salina
Halothece sp. PCC 7418
Synechococcus sp. PCC 8807
Leptolyngbya sp. PCC 7376
Limnothrix rosea
Cyanobacterium aponinum
Geminocystis sp. NIES 3708
Geminocystis herdmanii
Geminocystis sp. NIES 3709
Crinalium epipsammum
Leptolyngbya sp. JSC 1
Leptolyngbya sp. O 77
Oscillatoriales cyanobacterium MTP1
Geitlerinema sp. PCC 7407
Oscillatoriales cyanobacterium JSC 12
Prochlorothrix hollandica
Leptolyngbya sp. hensonii
Phormidesmis priestleyi
Leptolyngbya boryana
Leptolyngbya sp. NIES 2104
Leptolyngbya sp. NIES 3755
Desertifilum sp. IPPAS B 1220
Synechococcus sp. PCC 7335
Leptolyngbya sp. Heron Island J
Leptolyngbya sp. PCC 7375
Leptolyngbya sp. PCC 6406
Lyngbya confervoides
Halomicronema hongdechloris
Leptolyngbya sp. KIOST 1
Phormidium tenue
Nodosilinea nodulosa
Oscillatoria sp. PCC 10802
Oscillatoria acuminata
Planktothricoides sp. SR001
Geitlerinema sp. PCC 9228
Geitlerinema sp. PCC 7105
Phormidium sp. HE10JO
Phormidium willei
Hydrocoleum sp. CS 953
Trichodesmium erythraeum
Kamptonema
Microcoleus vaginatus
Oscillatoria nigro viridis
Planktothrix paucivesiculata
Planktothrix serta
Planktothrix tepida
Arthrospira platensis
Lyngbya aestuarii
Lyngbya sp. PCC 8106
Phormidium ambiguum
Chroococcidiopsis thermalis
Aliterella atlantica
Chroogloeocystis siderophila
Gloeocapsa sp. PCC 7428
Calothrix rhizosoleniae
Richelia intracellularis
Rivularia sp. PCC 7116
Mastigocoleus testarum
cyanobacterium PCC 7702
Chlorogloeopsis fritschii
Fischerella sp. PCC 9605
Fischerella muscicola
Fischerella thermalis
Fischerella sp. PCC 9431
Mastigocladus laminosus
Scytonema hofmanni. UTEX B 1581
Tolypothrix sp. NIES 4075
Calothrix sp. 336 3
Calothrix parietina
Calothrix sp. HK 06
Calothrix sp. PCC 7103
Scytonema hofmannii
Tolypothrix bouteillei
Mastigocladopsis repens
Nostocales cyanobacterium HT 58 2
Scytonema tolypothrichoides
Scytonema sp. HK 05
Tolypothrix campylonemoides
Cylindrospermum stagnale
Dolichospermum circinale
Anabaena sp. 90
Aphanizomenon flos aquae
Anabaena sp. PCC 7108
Trichormus sp. NMC 1
Anabaena cylindrica
Trichormus azollae
Cylindrospermopsis raciborskii
Nostoc piscinale
Nostoc sp. PCC 7107
Nodularia sp. NIES 3585
Nodularia spumigena
Nostoc sp. PCC 7524
Nostoc sp. NIES 3756
Nostoc sp. PCC 7120
Nostoc calcicola
Nostoc punctiforme
Nostoc sp. KVJ20
Nostoc sp. Peltigera membranacea cyanobiont. 232
Calothrix sp. PCC 7507
Fortiea contorta
Tolypothrix sp. PCC 7601
Nostoc sp. RF31Y
Nostoc sp. T09
0.3

Fig. S6

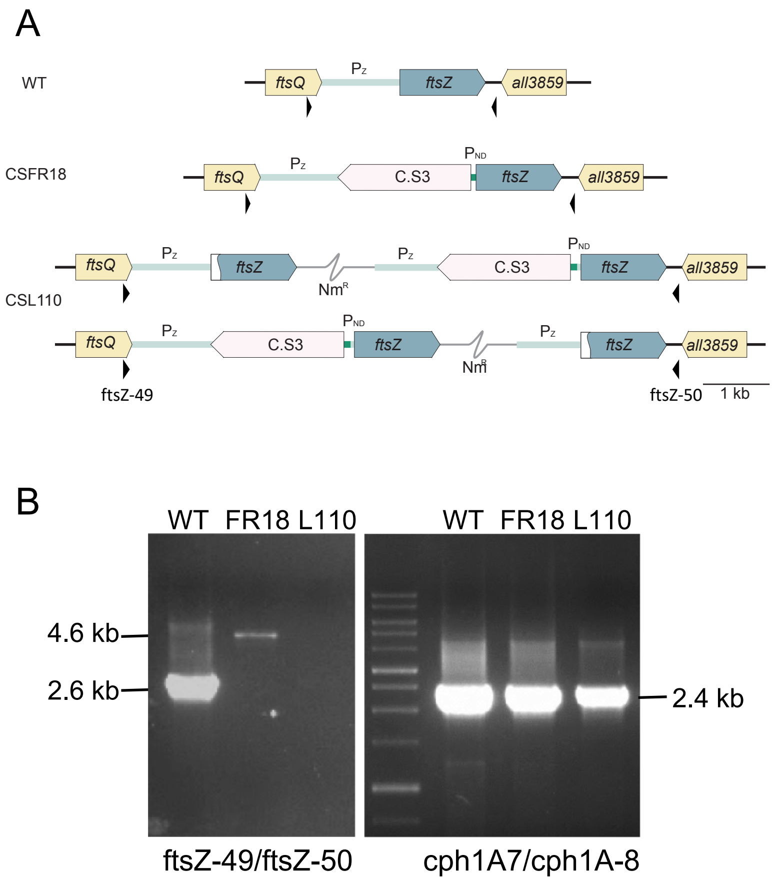
A
WT
ftsQ
Pz
ftsZ
all3859
CSFR18
C.S3
PND
CSL110
NmR
ftsZ-49
ftsZ-50
1 kb
B
WT FR18 L110
WT FR18 L110
4.6 kb
2.6 kb
2.4 kb
ftsZ-49/ftsZ-50
cph1A7/cph1A-8

Fig. S7

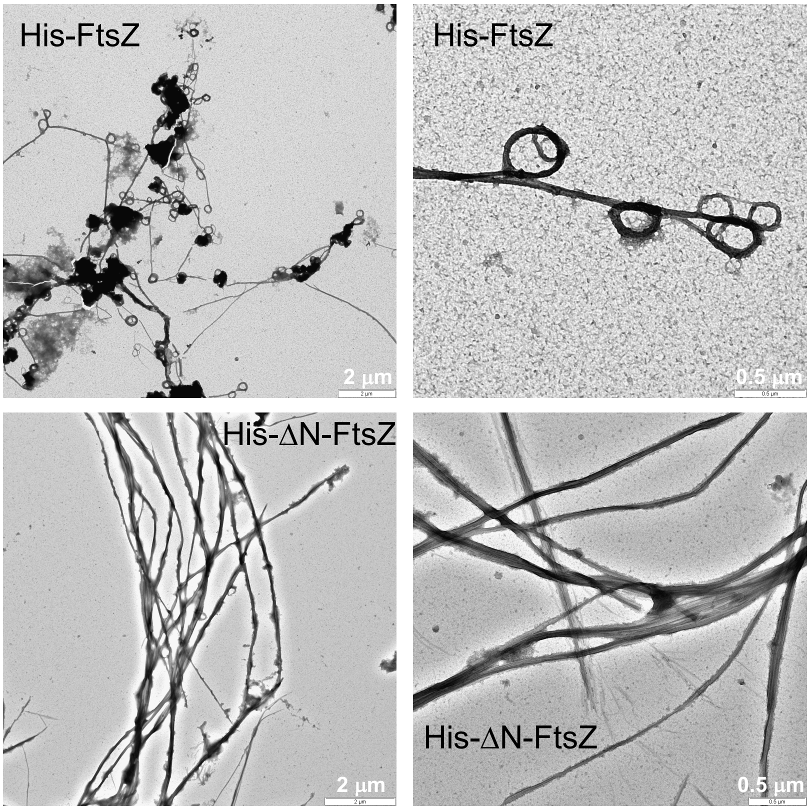

Fig. S8

A

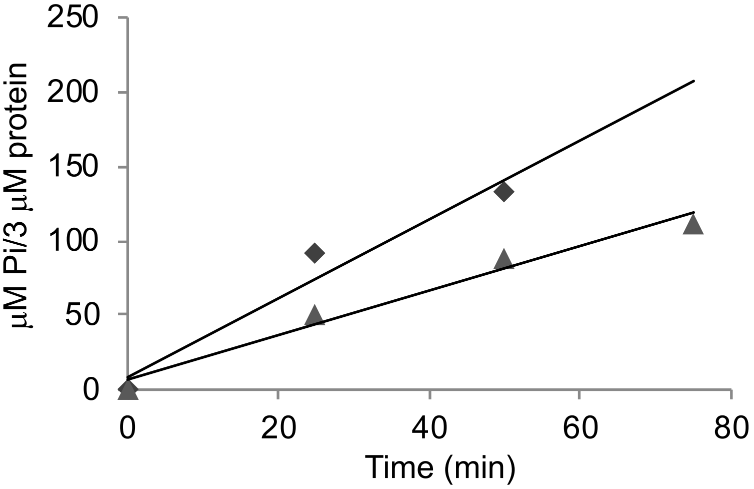

B

	GTPase activity (μM Pi.min^{-1}.μM protein^{-1}) 50 mM KCl	300 mM KCl
His-FtsZ	0.83	0.76
His-ΔN-FtsZ	0.53	0.42

Fig. S9

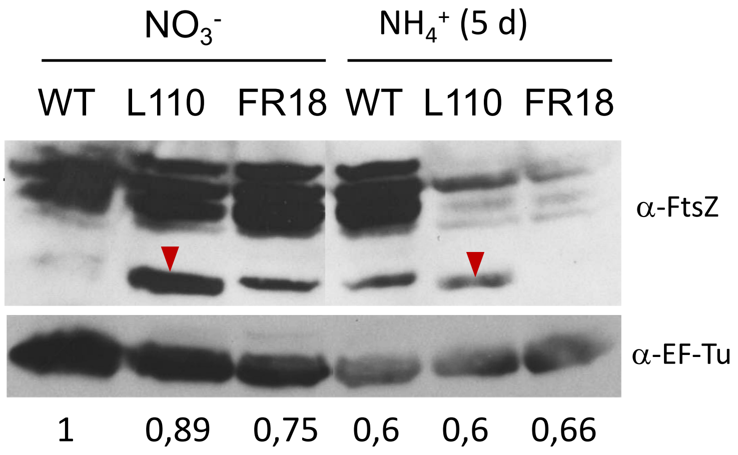

NO3-
NH4+ (5 d)
WT L110 FR18 WT L110 FR18
α-FtsZ
α-EF-Tu
1 0,89 0,75 0,6 0,6 0,66

Legends for Supplementary Figures

Fig. S1. FtsZ tree (top left); alignment of FtsZ N-terminal region (top right) and sequence logos (botton right) from an archaea (*Methanocaldococcus*), *Bacillus subtilis*, *Escherichia coli*, five Melainabacteria (*Obscuribacter*, *Gastranaerophilaceae* and *Melainabacteria*) and its sister phylum Cyanobacteria. In the phylogenetic tree, colors identify different phylogenetic clades (as in Figs. 1 and 2). Scale represents genetic distance. In the alignment, gaps are colored white whereas a residue is colored dark blue if it matches the consensus sequence at that position. If it does not match the consensus residue but the 2 residues have a positive Blosum62 score, it is colored light blue. In sequence logos, the height of each letter (vertical axis) is proportional to its frequency, the most common one are on top, whereas the horizontal axis represents the position of residues used in the analysis.

Fig. S2. Phylogeny of cyanobacteria based on 97 concatenated proteins. Colors indicate different phylogenetic clades as in Fig. S1. Scale represents genetic distance.

Fig. S3. Phylogeny of cyanobacteria based on the concatenation of 16S and 23S rRNA sequences. Colors indicate different phylogenetic clades as above. Scale represents genetic distance.

Fig. S4. Phylogenetic reconstruction of MinC in the phylum cyanobacteria. Analysis of a representative set of 97 cyanobacteria. Colors indicate different phylogenetic clades as above. Scale represents genetic distance.

Fig. S5. Phylogenetic reconstruction of a representative set of 146 ZipN sequences in the phylum cyanobacteria. Colors indicate different phylogenetic clades as above. Scale represents genetic distance.

Fig. S6. Segregation of strain CSL110. (A) genomic *ftsZ* region in *Anabaena* (WT) and strains CSL110 (L110) and CSFR18 (FR18). (B) PCR was performed with total genomic DNA isolated from BG11 cultures and the indicated oligonucleotide pairs. The position of oligonucleotides of the *ftsZ* region are represented in (A). With ftsZ-49/ftsZ-50, the absence of a 4.6 kb band in strain CSL110 implies its lacking of CSFR18-type chromosomes. PCR with cph1A-7/cph1A-8 of the *cph1* genomic region was used as a control for DNA quality.

Legends for Supplementary Figures (continued)

Fig. S7. Polymers of His-tagged *Anabaena* FtsZ and ΔN-FtsZ proteins. Proteins (3 μM) were incubated in the presence of 2 mM GTP under the conditions specified under Materials and methods during 20 min, and visualized after staining with uranyl acetate. Magnification was: 2,000x (upper left panel), 2,500x (lower left panel), 10,000x (upper right panel), 8,000x (lower right panel).

Fig. S8. GTPase activity of His-tagged *Anabaena* FtsZ and ΔN-FtsZ proteins. A. GTPase activity was determined with 3 μM of 6His-FtsZ (diamonds) or 6His-ΔN-FtsZ (triangles). At the times indicated, Pi produced was determined in aliquots of each reaction mixture. B. GTPase activity was determined in reaction mixtures containing 3 μM protein and 50 or 300 mM KCl. Figures are the mean of two independent assays with very similar results.

Fig. S9. Additional western blot analysis of FtsZ. BG11-grown filaments of *Anabaena* (WT) and strains CSL110 (L110) and CSFR18 (FR18) incubated for 5 days in $BG11_0$ plus ammonium (NH_4^+) or in nitrate-containing BG11 (NO_3^-) medium were used to prepare cell extracts. Aliquots of the resulting preparations were loaded into 15 % SDS/PAGE gels, electrophoresed and probed with antibodies raised against the *Anabaena* FtsZ protein (upper panel). As a loading control, hybridization was also performed with an antibody against the EF-Tu factor (relative amounts obtained after scanning and quantification are indicated) (lower panel). Red arrowheads point to a putative ΔN-FtsZ band.
