# Supplementary material for: FtsZ of Filamentous, Heterocyst-Forming Cyanobacteria Has a Conserved N-Terminal Peptide Required for Normal FtsZ Polymerization and Cell Division
Source: Front Microbiol. 2018 Oct 2;9:2260. doi: 10.3389/fmicb.2018.02260 (PMC6175996; doi:10.3389/fmicb.2018.02260)
Supplement: Supplementary file 2 [file Data_Sheet_2.PDF]

Supplementary text

Congruence among different phylogenetic approaches

Deep phylogenetic relationships are commonly established by reconstructing phylogenies from a dataset of concatenated proteins or the concatenation of large and small ribosomal genes. Recent attempts to reconstruct evolutionary history of cyanobacteria involve the concatenation of i) 31 conserved proteins of 126 taxa *(Shih et al.*, 2013); ii) 97 proteins from 102 cyanobacterial genomes (Ponce-Toledo *et al*., 2017); iii) 23 conserved proteins from 191 genomes (Mareš, 2017); iv) the genes for small and large ribosomal subunits of 96 most diverse taxa (after removing overrepresented strains such as *Prochlorococcus* and *Microcystis*) (Santamaria-Gomez *et al*., 2016). These independent approaches agree in the recovery of main clusters (colored in the figures), in the order of divergence of most clusters as well as the position of early diverging strains among each cluster (i.e. *Spirulina* and *Rubidibacter* for red group and *Rivularia* for light blue group heterocystous). These trees are also congruent with our phylogenetic reconstructions based on concatenated datasets of proteins (Fig. S2), rRNAs (Fig. S3), and single gene phylogenies of FtsZ (Fig. 2), MinC (Fig. S4) and ZipN (Fig. S5). Trees slightly differ in the evolutionary relationships among clusters as well as the position of very early divergent strains such as *Gloeomargarita lithophora*. The controversial position of this cyanobacterium is evidenced after comparing previous works in which *Gloeomargarita* diverges after Yellowstone *Synechococcus* strains (Couradeau *et al*., 2012, Moreira *et al*., 2017), after the *Pseudanabaena* cluster (Ponce-Toledo *et al*., 2017), or after the cluster containing *Thermosynechococcus* (Sanchez-Baracaldo *et al*., 2017). These disparate positions suggest that *Gloeomargarita* sequences could violate model specifications (compositional bias, rate heterogenety, among others).

Noteworthy, all trees mentioned above share important features that we consider for our study: i) Yellowstone strains diverge after *Gloeobacter*; ii) red and light blue clusters are monophyletic; iii) early diverging strains of each cluster are included in the phylogenetic reconstruction (providing a reliable common mark for the diversification starting point of each lineage). These features allow a quantitative estimation of the relative contribution of a lineage expansion to genetic protein divergence with respect to the changes accumulated since a reference point (the branching point of Yellowstone *Synechococcus* linage).

Couradeau, E., Benzerara, K., Gerard, E., Moreira, D., Bernard, S., *et al.* (2012) An early-branching microbialite cyanobacterium forms intracellular carbonates. *Science* **336**: 459-62. doi: 10.1126/science.1216171.

Mareš, J. (2017) Multilocus and SSU rRNA gene phylogenetic analyses of available cyanobacterial genomes, and their relation to the current taxonomic system. *Hydrobiologia*. doi: 10.1007/s10750-017-3373-2.

Moreira, D., Tavera, R., Benzerara, K., Skouri-Panet, F., Couradeau, E., *et al.* (2017) Description of *Gloeomargarita lithophora* gen. nov., sp. nov., a thylakoid-bearing, basal-branching cyanobacterium with intracellular carbonates, and proposal for Gloeomargaritales ord. nov." *Int J Syst Evol Microbiol* **67**: 653-658. doi: 10.1099/ijsem.0.001679.

Ponce-Toledo, R.I., Deschamps, P. Lopez-Garcia, P., Zivanovic, Y., Benzerara, K., and Moreira, D. (2017) An early-branching freshwater cyanobacterium at the origin of plastids. *Curr Biol* **27**: 386-391. doi: 10.1016/j.cub.2016.11.056.

Sanchez-Baracaldo, P., Raven, J.A., Pisani, D., and Knoll, A.H. (2017 Early photosynthetic eukaryotes inhabited low-salinity habitats. *Proc Natl Acad Sci USA* **114**: E7737-E7745. doi: 10.1073/pnas.1620089114.

Santamaria-Gomez, J., Ochoa de Alda, J.A.G., Olmedo-Verd, E., Bru-Martinez, R., and Luque, I. (2016) Sub-cellular localization and complex formation by aminoacyl-tRNA synthetases in cyanobacteria: Evidence for interaction of membrane-anchored ValRS with ATP synthase. *Front Microbiol* **7**: 857. doi: 10.3389/fmicb.2016.00857.

Shih, P. M., Wu, D., Latifi, A., Axen S. D., Fewer, D.P., *et al*. (2013) Improving the coverage of the cyanobacterial phylum using diversity-driven genome sequencing. *Proc Natl Acad Sci USA* **110**: 1053-8. doi: 10.1073/pnas.1217107110.
