# Supplementary material for: FtsZ of Filamentous, Heterocyst-Forming Cyanobacteria Has a Conserved N-Terminal Peptide Required for Normal FtsZ Polymerization and Cell Division
Source: Front Microbiol. 2018 Oct 2;9:2260. doi: 10.3389/fmicb.2018.02260 (PMC6175996; doi:10.3389/fmicb.2018.02260)
Supplement: Supplementary file 3 [file Data_Sheet_3.PDF]

Table S1. Oligonucleotide primers used in this work

ftsZ-8	TAAACTCGAGTTAATTTTTGGGTGGT
ftsZ-15	GACTTTGAGCTCGAACAGCTTGATAGGCTT
ftsZ-16	GCCAATACGGTTATTCATTGGATTCGGGAA
ftsZ-17	TTCCCGAATCCAATGAATAACCGTATTGGC
ftsZ-18	CACACCGAGCTCCATCAATGCTGATCCCG
ftsZ-19	GACTTTGGTACCGAACAGCTTGATAGGCTT
ftsZ-20	ATCTAAGGTACCTTAATTTTTGGGTGGT
ftsZ-24	CCTCCCCGTATCCAATGAATA
ftsZ-25	TCAGATTCAATCATGCGGTT
ftsZ-28	ATCCAATGACACTTGATAAGAACCAA
ftsZ-29	TCTTCTTACTGTCGTTATTTTGTCCAA
ftsZ-31	GATTTCTGCAGAAAATAACCGTATT
ftsZ-32	GTGAATTCTTATTAATTTTTGGGTGGTC
ftsZ-33	GATTTCCTGCAGAAATAACCGTATT
ftsZ-34	CCGAATCCCTGCAGACTTGATAATAAC
ftsZ-35	CCGAATCCTGCAGCACTTGATAATAAC
ftsZ-36	GATAGAGCTCCTGATTGACTTTACGACTG
ftsZ-37	ATCTGCTAGC**ACCTCCACCGCC**ATTTTTGGGTGGTCGCCGTC
ftsZ-38	GAGGAGCTCACCGAACAGCTTGATAG
ftsZ-39	CGGGCTAGC**ACCTCCACCGCC**ATTTTTGGGTGGTCGCCGTC
ftsZ-43	GCCACACTTGATAATAACCAA
ftsZ-44	GCCAATAACCGTATTGGCGAG
ftsZ-45	AAAACCGAATTCTTTTTGGGTGGTCGCCGTCTCT
ftsZ-46	GAATCCCTGCAGACTTGATAATAACCAAGAG
ftsZ-47	CTTTGCCACTTCCGCAACA
ftsZ-48	AGTCGTTTATCTACCTTCCCGAATC
ftsZ-49	ATCCTGAATCGCCTTTAGTACA
ftsZ-50	CATTTAGCCTGACTAGAGACT
cph1A-7	GCGATCGCTTCCATCTGTACCAAA
cph1A-8	TGACTTACTGAAGCCCGGAAGGTT
alr0599-1	CCAAATAGCTGGGCCAGTGTTAGT
alr0599-2	GGAATTGCTTTGCCAGTTGTCAG
all5167-1	GCTCAAGCAATTCGTCACTGTTCC
all5167-2	AAAGATTGCGTCGGTCTGGTGT
minC-1	CATGCCTGCAGTTCTGATTCTGCC
minC-2	CATGCCTGCAGCTTCTGATTCTGCC
minC-3	TAAAGGTACCAATTATTATGGTGTCTGATT
minC-4	TAAAGGTACCAATGGTGTCTGATT
zipN-21	AATAAACTGCAGTGATCACGGTGCAGG
zipN-22	CAAAAAGGTACCCTTAATTTATAGCGGCTGAC
sepF-16	AGTGCACTGCAGTGAACAATATATTTTCTAAAC
sepF-17	GACTAAGAATTCTTTATTGTGCCATCCGGT
sepF-18	GTGCAACTGCAGGAACAATATATTTTCTAAAC
sepF-21	ATTTAAGAATTCTGTGCCATCCGGTTGGTTTC

Introduced restriction sites and a sequence encoding a 4Gly-linker are denoted underlined and in bold, respectively.
