# Supplementary material for: FtsZ of Filamentous, Heterocyst-Forming Cyanobacteria Has a Conserved N-Terminal Peptide Required for Normal FtsZ Polymerization and Cell Division
Source: Front Microbiol. 2018 Oct 2;9:2260. doi: 10.3389/fmicb.2018.02260 (PMC6175996; doi:10.3389/fmicb.2018.02260)
Supplement: Supplementary file 4 [file Data_Sheet_4.PDF]

Table S2. FtsZ interactions by BACTH analysis

	T18-FtsZ	T18-ΔN-FtsZ	T18-SepF	T18-MinC	pUT18C		FtsZ-T18	ΔN-FtsZ-T18	SepF-T18	MinC-T18	pUT18
**T25-FtsZ**	16.1±1.6 (4)	14.4±4.6 (6)	15.0±4.5 (8)	**19.4±3.0** (7)*	14.5±2.9 (5)		**74.9±9.1** (5)**	**52.8±5.6** (5)**	21.4+1.3 (3)	19.0±1.9 (7)	16.3±3.0 (5)
**T25-ΔN-FtsZ**	16.4±3.4 (5)	**22.3±1.7** (4)**	16.3±2.8 (5)	**21.6±2.6** (7)**	15.7±1.7 (4)		**86.7±8.3** (6)**	**63.6±9.9** (4)**	19.2±2.0 (4)	**21.0±2.3** (7)**	13.3±2.6 (4)
**T25-ZipN**	**31.6±4.5** (5)**	**33.2±2.5** (5)**			12.3±1.2 (3)		**32.3±2.2** (3)*	16.0±1.3 (3)			10.9±2.4 (3)
**T25-SepF**	16.5±5.7 (5)	11.3±2.6 (4)			8.5±0.5 (3)		**23.8±4.3** (3)*	17.0±2.3 (3)			9.6±2.0 (3)
**T25-MinC**	19.3±1.8 (7)	21.9±1.3 (7)			18.4±6.7 (4)		21.1±4.0 (4)	18.5±1.0 (4)			18.0±5.3 (4)
**pKT25**	12.9±3.2 (7)	12.2±4.7 (8)	9.2±0.5 (3)	12.1±3.1 (3)	17.4±0.4 (4)		16.2±2.6 (6)	15.1±1.0 (6)	20.6±2.8 (3)	14.0±0.0 (2)	18.2±1.7 (4)
**FtsZ-T25**	**60.6±9.0** (10)**	**75.9±16.0** (9)**	13.7±2.6 (5)	23.5±3.9 (5)	20.0±2.5 (4)		**60.9±5.6** (5)**	**27.4±1.6** (4)**	**49.2±2.8** (3)**	24.0±4.9 (8)	17.9±4.4 (6)
**ΔN-FtsZ-T25**	**41.8±7.4** (4)**	**53.3±9.2** (4)**	13.4±3.8 (5)	18.2±0.6 (4)	17.7±1.8 (4)		**23.2±1.4** (5)**	16.3±2.2 (6)	19.9±1.7 (4)	17.7±1.8 (4)	15.7±2.0 (4)
**SepF-T25**	19.4±2.9 (3)	20.3±2.1 (4)			14.7±3.3 (3)		**44.9±6.7** (4)**	19.4±1.8 (4)			17.8±3.7 (3)
**MinC-T25**	20.8±2.5 (7)	24.5±6.1 (9)			18.1±6.7 (4)		**25.1±3.8** (4)*	17.4±2.2 (4)			16.8±5.1 (4)
**pKNT25**	11.8±3.2 (12)	11.5±3.8 (10)	9.1±0.7 (3)	18.8±6.8 (4)	18.7±0.6 (4)		18.3±2.3 (4)	14.6±2.7 (6)	20.1±1.2 (3)	19.1±4.4 (4)	17.2±1.2 (4)

The topology of the fusion is indicated by the order of components (T18-protein and T25-protein denotes the corresponding adenylate cyclase domain fused to the N-terminus of the tested protein; protein-T18 and protein-T25 denotes the corresponding adenylate cyclase domain fused to the C-terminus of the tested protein). Values (nmol ONPG$\cdot$min$^{-1}\cdot$mg prot^{-1}) are given as mean±standard deviation (number of assays with independent transformants performed); boldface type denotes significant differences as assessed by Student's *t* tests (*p<0.05; **p<0.01; values with regard to the two respective controls: bacteria containing one of the two plasmids encoding a fused gene and the complementary empty vector). Positive control, SepJ-T18/T25-FtsQ (see Ramos-León *et al.*, 2015): 88±16.5 (5).
